# Dietary Polyphenols in Type 2 Diabetes: A Metabolite-Centric Review of Human Evidence

**DOI:** 10.3390/nu18172856

**Published:** 2026-09-01

**Authors:** Celia María Curieses Andrés, José Manuel Pérez de la Lastra, Elena Bustamante Munguira, Celia Andrés Juan, Eduardo Pérez Lebeña

**Affiliations:** 1 Hospital Clínico Universitario de Valladolid, Avenida de Ramón y Cajal, 3, 47003 Valladolid, Spain; cmcuriesesa@saludcastillayleon.es (C.M.C.A.);; 2 Institute of Natural Products and Agrobiology, CSIC-Spanish Research Council, Avda. Astrofísico Fco. Sánchez, 3, 38206 La Laguna, Spain; 3 Cinquima Institute and Department of Organic Chemistry, Faculty of Sciences, Valladolid University, Paseo de Belén, 7, 47011 Valladolid, Spain; celia.andres.juan@uva.es; 4 Sistemas de Biotecnología y Recursos Naturales, 47625 Valladolid, Spain; info@glize.eu

**Keywords:** type 2 diabetes, dietary polyphenols, anthocyanins, resveratrol, green tea catechins, cocoa flavanols, olive phenolics, gut microbiome catabolites, meal timing

## Abstract

Dietary polyphenols are studied as potential adjuncts for type 2 diabetes (T2D), although many reviews still rely on an antioxidant framework that does not adequately reflect human exposure. This review organises the evidence around two metabolite waves, namely early postprandial phase-II conjugates produced by host enzymes within 0 to 4 h of a polyphenol-rich meal, and microbiome-derived catabolites including urolithins and γ-valerolactones that peak at 6 to 24 h. This biphasic interpretation is advanced as a hypothesis-generating framework rather than as an established explanation, and it offers a testable account of why polyphenol effects may be meal-contingent, metabotype-dependent and heterogeneous across clinical trials. Using randomised controlled trials and meta-analyses published between 2015 and 2025, we review the human evidence for anthocyanins, catechins, stilbenes, cocoa flavanols and olive phenolics, with briefer coverage of isoflavones, curcuminoids and ellagitannins. Anthocyanins show the most consistent glycaemic signal, with a best available pooled estimate of about 0.3% for HbA1c (−0.31%, median exposure eight weeks) derived from trials that did not verify metabolite exposure, an effect that is modest and lies at the lower bound of what is generally regarded as clinically meaningful. Green tea catechins and high-phenolic extra-virgin olive oil are associated with comparable postprandial gains, whereas cocoa flavanols and resveratrol more reliably improve vascular and inflammatory intermediates than glycaemia itself. Most interventions were well tolerated at the nutritional doses tested. Polyphenols are better regarded as meal-timed dietary adjuncts to standard T2D care than as primary glycaemic agents.

## 1. Introduction

Type 2 diabetes (T2D) continues to be a major clinical challenge, and its management has expanded well beyond glucose control alone towards organ protection, realistic remission in selected patients and explicit recognition of coexisting liver disease. The reclassification of non-alcoholic fatty liver disease as metabolic dysfunction-associated steatotic liver disease (MASLD) anchors hepatic disease within the metabolic syndrome framework, reshaping clinical endpoints towards cardiovascular, renal and glycaemic risk [1].

Mechanistically, T2D is multisystemic, since mitochondrial injury involving cytosolic mtDNA release activates cGAS-STING and sustains low-grade inflammation, while emerging data on β-cell senescence and dedifferentiation link metabolic stress to innate immunity and cellular ageing [2].

Clinical interest in dietary polyphenols as adjuncts for T2D is substantial, although the traditional antioxidant model is now too limited. Contemporary redox biology distinguishes eustress, understood as localised and informational oxidant pulses, from distress, understood as damaging excess, which explains why broad antioxidant supplementation consistently falls short and opens space for signal-modulating strategies instead [3].

A more informative starting point is the set of metabolites that tissues actually encounter. Following a polyphenol-rich meal, intestinal and hepatic enzymes rapidly generate glucuronides, sulphates and O-methylated conjugates, which transporters then route towards mucosal and canalicular surfaces while plasma aglycone levels remain negligible throughout [4]. Some hours later, the gut microbiome converts unabsorbed precursors into urolithins, γ-valerolactones and phenylacid derivatives, which circulate as conjugates and reach extra-intestinal tissues [5]. This biphasic exposure profile helps determine the reach, timing and biological intensity of polyphenol effects in humans.

This model also helps account for heterogeneity, since metabotype status, meaning who produces urolithins or equol, together with transporter genetics, background diet, and medication use, all influence tissue exposure, so identical intakes can produce markedly divergent responses across individuals [6].

**Figure 1:**
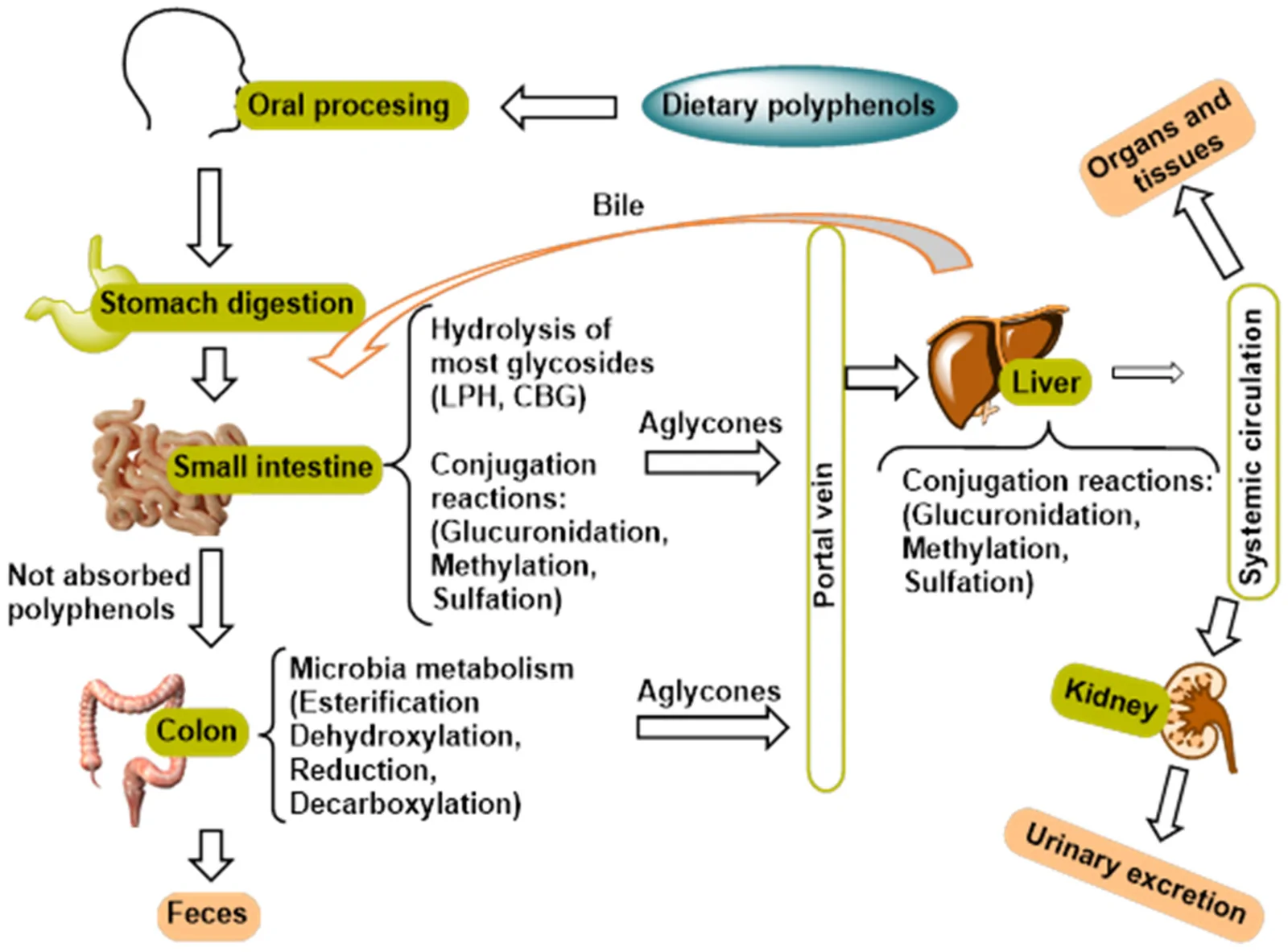
Absorption and metabolic pathways of dietary polyphenols and their derivatives in humans.

Figure 1 summarises the principal absorption and metabolic routes followed by dietary polyphenols and their derivatives once they are ingested by humans. It traces the pathway from initial intestinal uptake through hepatic and microbial biotransformation, showing how these successive steps generate the diverse circulating metabolites that eventually reach peripheral tissues.

One issue requires particular care. The evidence that time-restricted eating aligned with morning hours improves HbA1c and glycaemic variability in T2D is now substantial [7], whereas the inference that timing polyphenol intake to earlier meals specifically amplifies metabolite exposure and glycaemic benefit is mechanistically well-grounded but rests on indirect evidence rather than on dedicated polyphenol timing trials. We therefore make this distinction explicit throughout the review.

We use a narrative review design centred on metabolite exposure, and we state at the outset that the two-wave model is offered as a hypothesis-generating framework whose clinical validation is still outstanding. Its mechanistic derivation was set out by our group in a previous publication [8], and the present review is intended as its clinical companion, confined to human clinical evidence in T2D. We prioritise human randomised trials and, where available, meta-analyses, and we emphasise verified metabolite exposure, clinically anchored endpoints and timing-sensitive designs as the basis for more reproducible progress.

## 2. Scope and Structure of the Review

This review covers the human evidence linking dietary polyphenols with T2D through a metabolite-focused perspective, drawing mainly on randomised trials in adults with T2D and, where informative, in adults with prediabetes. We give particular weight to studies that verify authentic metabolite exposure across the early window of 0 to 4 h and the late window of 6 to 24 h following a meal. The outcomes considered are HbA1c, fasting and postprandial glycaemia, indices of insulin resistance and sensitivity and CGM metrics, together with hepatic, vascular, inflammatory, adherence and safety data, given the substantial overlap between T2D and MASLD.

We deliberately chose a narrative design rather than a fully systematic format, because pooling trials that differ so widely in compound, dose, matrix, duration, metabolite verification status and background therapy could yield estimates that look precise but are biologically misleading. The review question, the search strategy and the eligibility criteria were nonetheless defined in advance, and findings are synthesised narratively within each class. We conduct no original pooling of any kind, and every quantitative summary reported here is taken from a previously published meta-analysis that we cite at the point of use.

## 3. Methods

This is a narrative review with a structured search strategy and it is not a systematic review. We have therefore not registered a protocol, and we report neither PRISMA 2020 items, nor a study-selection flow diagram, nor record counts by stage, nor a formal risk-of-bias instrument, so readers should weigh the synthesis accordingly. We defined the review question and the primary outcomes in advance, conducted systematic database searches and applied prespecified eligibility criteria. Evidence is synthesised narratively by polyphenol class and weighted by study quality and exposure verification status, and where published meta-analyses already provide robust pooled data we cite and interpret those findings rather than repeating the pooling ourselves.

### 3.1. Search Strategy

We searched MEDLINE through PubMed (https://pubmed.ncbi.nlm.nih.gov/, accessed on 14 October 2025), together with Embase (https://www.embase.com/, accessed on 14 October 2025), the Web of Science Core Collection (https://www.webofscience.com/, accessed on 14 October 2025) and Scopus (https://www.scopus.com/, accessed on 14 October 2025), and we supplemented these with Cochrane CENTRAL (https://www.cochranelibrary.com/central, accessed on 14 October 2025) and the ClinicalTrials.gov (https://clinicaltrials.gov/, accessed on 14 October 2025) and EU Clinical Trials (https://www.clinicaltrialsregister.eu/, accessed on 14 October 2025) registries, covering the period from 1 January 2015 to 14 October 2025 [9]. Search strings combined controlled vocabulary for T2D and the relevant polyphenol classes with outcome terms and filters for randomised trials and meta-analyses, and they also included xenometabolite terms such as urolithin, γ-valerolactone and glucuronide in order to capture trials with verified exposure. Reference lists and forward citations were searched by hand, and the full database-specific strings, together with the dates on which each search was last run, are available from the corresponding author on request.

### 3.2. Eligibility Criteria

Population. Adults aged eighteen years or older with established T2D, defined using fasting glucose, OGTT or HbA1c criteria. Trials conducted in people with prediabetes were also eligible, although they were analysed separately and are identified as such wherever they are cited. Evidence drawn from other populations, including metabolic syndrome, overweight or obesity, MASLD without diabetes and healthy adults, is used only to establish biological plausibility and is labelled as indirect evidence at every point of use. Type 1, monogenic and gestational diabetes were excluded, as were participants with acute illness or uncontrolled comorbidity, while stable background therapies were permitted provided that these remained balanced across the trial arms.

Interventions. Dietary polyphenols delivered as whole foods, enriched products, standardised extracts or isolated compounds, spanning anthocyanins, stilbenes and resveratrol, catechins and green tea, cocoa flavanols and olive phenolics. Trials were required to report both dose and polyphenol class; particular priority was given to those verifying phase-II conjugates or microbiome catabolites in plasma or urine, and multi-ingredient formulations were excluded whenever the polyphenol-specific effect could not be separated from other components.

**Comparators.** Placebo, low-polyphenol controls, energy or matrix-matched controls, and usual diet were all considered acceptable. Crossover designs were only considered eligible when they included an adequate washout period between arms.

Outcomes. The primary outcomes considered were HbA1c, fasting and postprandial glucose including OGTT-derived indices, HOMA-IR and the Matsuda index. Secondary outcomes included CGM metrics such as time-in-range, mean glucose and glycaemic variability, together with cardiometabolic intermediates, hepatic endpoints such as ALT, AST and MRI-PDFF, validated inflammatory and redox markers, safety data, adherence and metabolite verification data.

**Design and time frame.** We included randomised controlled trials, whether parallel or crossover, together with systematic reviews and meta-analyses. We required a minimum follow-up of eight weeks or more for HbA1c and of two to four weeks for fasting or postprandial indices. Acute testing was used only to inform mechanistic understanding, rather than to establish clinical efficacy.

### 3.3. Study Selection and Data Extraction

Screening was conducted in two sequential stages. Obvious exclusions, including animal or in vitro studies, single-meal tests without clinical endpoints, paediatric populations, gestational or type 1 diabetes, and multi-ingredient products with inseparable effects, were removed at the title and abstract stage, and the remaining records then underwent full-text review. Screening was carried out by one author and checked by a second, rather than performed independently in duplicate, and any disagreements were resolved by discussion. We record this explicitly because it is a departure from systematic-review practice and it constrains the reliability of study selection. Records were de-duplicated by DOI, author, title and year before screening began.

Data extraction relied on a piloted, standardised form that captured trial design and registration details, participant characteristics, intervention details such as polyphenol class, matrix, dose, formulation and timing, exposure verification data including the analyte panel, matrices and sampling windows, and outcomes together with adherence and adverse events. Because no original pooling was undertaken, no variance imputation, control-group apportionment or paired-estimate handling was required. Effect estimates are reported as they appear in the source publication, together with that publication’s own description of the contributing trials.

### 3.4. Study Quality Appraisal

We appraised study quality qualitatively, using criteria appropriate to each design, and we did not apply Cochrane RoB 2, ROBINS-I or any other formal instrument, so no risk-of-bias table is presented. For randomised trials, we considered the adequacy of randomisation, blinding, comparator matching, duration and the handling of missing data; for crossover studies, we focused on washout adequacy and carryover; and for meta-analyses, we examined search scope, the management of heterogeneity and the assessment of publication bias. Food-based trials received additional scrutiny regarding organoleptic masking and energy and macronutrient matching, and metabolite-verified adherence was treated throughout as a marker of higher-quality evidence. We applied minimally important differences of approximately 0.3 to 0.4% for HbA1c, 5 to 10 mg/dL for fasting glucose and approximately five percentage points for CGM time-in-range when interpreting clinical relevance [10].

### 3.5. Evidence Synthesis

We synthesised the evidence across two complementary layers. The first layer consists of a narrative organised by polyphenol class, characterising design, dose, formulation, exposure verification status and clinical context, in order to expose the principal sources of heterogeneity. The second layer consists of quantitative summaries for continuous outcomes such as HbA1c, fasting and postprandial glucose, insulin resistance and CGM metrics, all of which are taken directly from previously published meta-analyses. To avoid any ambiguity, we performed no original quantitative synthesis, and the phrase compact meta-analyses used in an earlier version of this manuscript has been withdrawn. Each numerical estimate reported in this review is attributed to its source publication, and the population, exposure duration and metabolite-verification status of the contributing trials are stated alongside it. Inflammation and redox markers support the overall plausibility of our findings, although they do not drive quantitative conclusions unless paired with glycaemic endpoints.

### 3.6. Terminology for Evidence Certainty

Formal GRADE terminology has been withdrawn from this manuscript. We did not construct outcome-specific evidence profiles, and we did not make explicit judgements on risk of bias, inconsistency, indirectness, imprecision or publication bias, so the use of GRADE labels would have been unwarranted. In their place we apply a purely descriptive three-level confidence label. Consistent describes a direction of effect reproduced across several independent randomised trials of adequate duration with broadly comparable comparators. Probable describes a direction of effect reproduced in some trials but not others or resting mainly on secondary endpoints. Uncertain describes a body of evidence too small, too short, too heterogeneous or too indirect to support any directional statement. These labels carry no GRADE equivalence.

### 3.7. Figure Provenance

All figures in this manuscript are original works prepared by the authors, and none of them is reproduced or adapted from a copyrighted source, so no permission is required. The schematic figures were drawn with ChemDraw (Revvity Signals Software, Waltham, MA, USA; https://revvitysignals.com/products/research/chemdraw, accessed on 14 October 2025), and any structural or pathway content they depict is drawn from the primary literature cited in the corresponding section rather than reproduced from a published figure. The remaining figures are conceptual syntheses prepared by the authors, and each of them is identified as such in its caption. Those captioned as conceptual diagrams are not drawn to scale, since the curves, timings and group proportions they show are illustrative rather than derived from any dataset, model fit or measured distribution.

## 4. Pharmacokinetics and Real Exposure

After a polyphenol-rich meal, tissues do not mainly encounter the ingested aglycone itself, but rather two sequential metabolite waves. This biphasic pharmacology is presented throughout as a hypothesis-generating framework for interpreting trial results and for designing studies capable of detecting effects that current designs may miss. Its individual components are supported by human pharmacokinetic data, although the proposition that metabolite exposure in these two windows mediates clinical outcomes in T2D has not yet been tested directly.

The first wave occurs within 0 to 4 h and consists of host-derived phase-II conjugates, namely glucuronides, sulphates and O-methylated forms produced by UGTs, SULTs and COMT in enterocytes and hepatocytes [11]. Polarised ABC transporters, including MRP2, BCRP and P-gp, route these conjugates preferentially towards the intestinal lumen and bile canaliculi, so mucosal surfaces receive high local concentrations while systemic plasma levels remain comparatively low [12]. Local β-glucuronidases can transiently regenerate the aglycone at these interfaces, creating brief microbursts that activate redox sensors such as Keap1 and IKKβ without any systemic aglycone exposure.

The second wave occurs between 6 and 24 h and arises from colonic microbial catabolism of unabsorbed or recirculated precursors. Urolithins from ellagitannins, γ-valerolactones from flavan-3-ols and procyanidins, and phenylacid or hippurate derivatives are smaller and more permeable molecules that circulate as conjugates and reach extra-intestinal tissues, engaging AMPK, TFEB-driven mitophagy and PPAR and AhR pathways [13].

This exposure pattern has three direct consequences for trial interpretation. Sampling only at an early time point misses the late metabolite wave entirely, so apparent null results at one to two hours post-dose may simply reflect an absence of measurement rather than true biological inactivity. The capacity to generate late-window catabolites varies considerably across individuals, giving rise to distinct metabotypes such as urolithin producers and non-producers, γ-valerolactone converters and equol producers, which together explain a large share of the heterogeneity observed between people. Finally, the timing of dosing relative to meals and time of day shapes which metabolite window predominates, and whether its peak coincides with periods of higher insulin sensitivity.

### 4.1. Absorption and Phase-II Metabolism

Most polyphenols are efficiently conjugated during first-pass metabolism in enterocytes and hepatocytes, which leaves the free aglycone essentially undetectable in systemic plasma even at relatively high intakes [14]. Regioselectivity matters, since UGT, SULT and COMT isoforms favour different hydroxyl positions [15] and thereby produce positional isomers that differ in charge, polarity, transporter affinity and signalling potential. Enzyme capacity also varies with dose, since PAPS-limited SULTs dominate at low intake and saturate early, directing higher loads towards glucuronidation and enterohepatic cycling [16], while common polymorphisms in UGT1A1, UGT1A9, SULT1A1 and COMT further amplify inter-individual differences [17]. These findings favour isomer-resolved LC-MS/MS analytics over bulk colorimetric assays and, where feasible, measurement of free rather than total concentrations.

### 4.2. Transporter-Mediated Tissue Distribution

Once phase-II conjugates predominate, their distribution across tissues is governed by polarised efflux and uptake transporters rather than by passive diffusion. At the intestinal epithelium, apical MRP2 (ABCC2) and BCRP (ABCG2) eject glucuronides and sulphates back into the lumen while P-gp (ABCB1) limits aglycone retention, so mucosal surfaces reach high local conjugate concentrations alongside low systemic exposure [18]. In the liver, canalicular transporters secrete conjugates into bile for enterohepatic recirculation, and when that capacity saturates, basolateral MRP3 back-exports conjugates into the systemic circulation without any change in intake [19].

Transporter expression is regulated by PXR, CAR, AhR and NRF2, and it remains sensitive to inflammatory states, hypoxia and common genetic variants including ABCG2 rs2231142 [20]. At apical and canalicular interfaces, microbial and immune β-glucuronidases cleave conjugates to regenerate the catechol, which activates Keap1 and IKKβ in a spatially confined manner before reconjugation limits its systemic spread [21].

Two methodological consequences follow. Total plasma polyphenol concentrations systematically underestimate tissue exposure at mucosal and biliary interfaces, so free fractions together with urine metabolite quantification give a considerably more complete picture. An apparent non-response may also reflect transporter-mediated redistribution rather than a true absence of exposure, and ruling this out requires simultaneous sampling of plasma and urine across both time windows.

### 4.3. Microbiome Catabolites: Urolithins, γ-Valerolactones, and Phenylacid Derivatives

The late metabolite wave differs from the early conjugate wave in its origin, structure and biological target. Ellagitannins from pomegranate, walnuts and certain berries are first hydrolysed to ellagic acid, then converted by colonic bacteria into urolithins A through D, which circulate predominantly as urolithin A-3-glucuronide [22]. Urolithin A activates mitophagy through the PINK1-Parkin pathway and promotes TFEB-driven lysosomal biogenesis, both processes known to be impaired in insulin-resistant skeletal muscle [23]. Urolithin production is metabotype-dependent, since population studies identify efficient producers, partial producers and non-producers, the last of whom make up approximately 25 to 40% of adults in Western populations and generate negligible urolithins even from identical intakes [24].

γ-Valerolactones arise from flavan-3-ols and procyanidins, found in green tea, cocoa and grape seeds, through microbial *C*-ring cleavage [25], chain shortening and subsequent lactonisation, producing 5-(hydroxyphenyl)-γ-valerolactone scaffolds that the host further O-methylates and conjugates. At low micromolar concentrations, these metabolites modulate AMPK and MAPK and interact with PPAR and AhR, linking energy sensing, inflammatory tone and lipid metabolism in a manner directly relevant to T2D [26]. The capacity for *C*-ring fission and lactonisation varies substantially across individuals [27], while phenylacid and hippurate derivatives are less class-specific and serve mainly as useful indices of overall microbiome engagement.

The trial-design implications are clear. Late-window sampling covering 8 to 12 h, in both plasma and spot urine, should be standard practice rather than an optional addition, and metabotype pre-screening with stratified randomisation is recommended for future trials rather than being treated as a formal methodological requirement, since pooling producers together with non-producers systematically attenuates any real effect that might otherwise be detected. A lean verification panel comprising urolithin A-3-glucuronide, a representative γ-valerolactone glucuronide and phenyl or hippurate markers is feasible in most trial settings and should be reported as a primary pharmacokinetic outcome. Table 1 summarises the two metabolite waves, together with their dietary precursor classes, metabotype classification and mechanistic relevance to type 2 diabetes.

### 4.4. Chrononutrition and Time-of-Day Effects

Insulin sensitivity, β-cell secretory efficiency and postprandial glycaemic control are consistently higher in the morning than in the evening, regardless of meal composition [28]. Controlled trials of early time-restricted eating reduce HbA1c, fasting glucose and glycaemic variability through mechanisms extending well beyond caloric restriction, and CGM studies show attenuated postprandial excursions for meals taken earlier in the day [29].

Two claims need to be separated clearly. The first, that earlier meal timing in general improves glycaemic control in T2D, is well supported by existing human evidence. The second, that timing polyphenol intake specifically to the first main meal enhances the metabolite exposure profile or the glycaemic benefit beyond what dose and formulation alone achieve, is mechanistically plausible but still lacks dedicated polyphenol timing trials. Most polyphenol RCTs standardise meal timing purely as a control condition rather than testing chrononutrition as an experimental variable, so we interpret timing recommendations as resting on mechanistic inference and indirect evidence, and we flag this limitation again in Section 9.5.

The pharmacokinetic rationale for meal-aligned dosing is quite distinct from the chrononutrition claim discussed above and remains strong. Polyphenol absorption and first-pass conjugation are tightly coupled to meal composition and gastric emptying, so dosing alongside a standard mixed meal containing fat and fibre produces a richer and earlier conjugate peak than dosing under fasted conditions [30]. Aligning the dose with the first main meal therefore exploits the higher morning insulin sensitivity window while also optimising early-window phase-II metabolism, and the late microbiome wave then peaks within a period that remains biologically active. Dosing time and meal timing should be standardised and explicitly reported, sampling should cover both metabolic windows, and any misaligned dosing should trigger dedicated sensitivity analyses.

### 4.5. Inter-Individual Variability: Metabotypes, Genetics, Diet, and Medications

The biological response to identical polyphenol intake varies substantially from one individual to another, and this variability reflects biological structure rather than random noise. The gut microbiome drives the most clinically consequential source of variability, acting through the metabotype system already described in Section 4.3. These patterns remain relatively stable within individuals over the course of months, although they can be modified by antibiotic use, PPI therapy, fibre intake and prolonged dietary change [31]. In trials lasting twelve weeks or more, re-screening metabotype status at the end of the trial is informative, since approximately 10 to 15% of participants may transition between classes over that period [32].

Host genetics add a second layer. Common variants in ABCG2, specifically rs2231142, and in ABCB1 alter efflux capacity and thereby shift tissue concentrations without any corresponding change in intake [33], while UGT, SULT and COMT polymorphisms similarly alter the dominant regioisomers produced at first pass. Local β-glucuronidase activity provides a third axis of variability, since inflamed or infiltrated tissues generate stronger local aglycone microbursts from the very same circulating conjugate pool, which may help explain why participants with MASLD or endothelial dysfunction sometimes show noticeably larger polyphenol responses.

Matrix effects are equally important in this context. Fat and emulsifiers enhance micellarisation and boost early-window conjugate flux, viscous fibre instead shifts substrate towards the late microbiome window, and protein can bind phenolics and reduce the bioavailable fraction [34], so comparators must be matched by macronutrient content rather than by sugar content alone. Medications also reshape the exposure landscape, since antibiotics can eliminate late-window catabolite generation for several weeks, PPIs alter gastric pH, transit time and the microbiome itself, and transporter inducers or inhibitors redirect conjugate distribution between compartments [35]. These drug effects should be carefully documented and treated as prespecified sources of variability, rather than merely listed among permitted background therapies.

**Figure 2:**
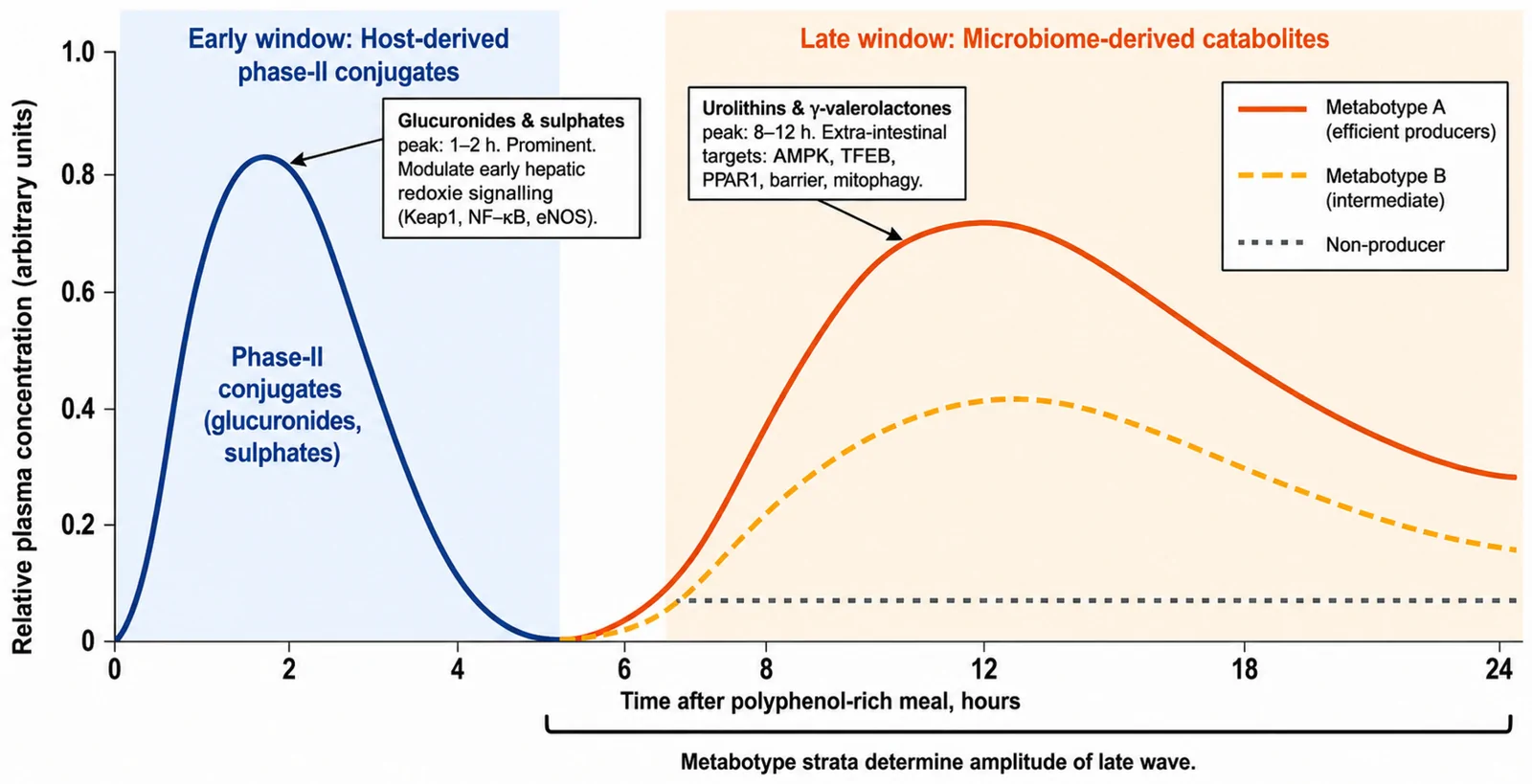
Conceptual diagram, not drawn to scale. Biphasic metabolite exposure profile following dietary polyphenol intake. Host-derived phase-II conjugates (glucuronides, sulphates) dominate the early window (0–4 h); microbiome catabolites (urolithins, γ-valerolactones) peak in the late window (6–24 h). Metabotype strata (A, B/mixed, non-producer) determine the amplitude of the late wave for ellagitannin-derived metabolites.

Figure 2 is a conceptual illustration of the pharmacokinetic rationale that underpins the metabolite-focused framework of this review, and the curves shown are schematic rather than derived from a pooled dataset or a fitted pharmacokinetic model. The early wave of host-derived phase-II conjugates peaks at approximately one to two hours and acts primarily at intestinal mucosal and hepatic canalicular interfaces, whereas the late wave of microbiome-derived urolithins and γ-valerolactones peaks between eight and twenty-four hours and reaches extra-intestinal targets including skeletal muscle, adipose tissue and the vasculature. The amplitude of that late wave is determined by individual metabotype, which is one reason why pooling participants without prior metabotype characterisation makes this literature difficult to interpret.

## 5. Clinical Evidence in T2D by Polyphenol Class

The estimates presented below, covering changes in HbA1c and fasting glucose, are taken from previously published meta-analyses or otherwise from individual trials within each polyphenol class. Each estimate is attributed to a named source at the point of use, and we state alongside it the population studied, the median or typical exposure duration, and whether the contributing trials verified metabolite exposure. Estimates derived from populations broader than adults with established T2D are stated explicitly and treated as indirect evidence. Effects are judged against clinically meaningful thresholds, namely around 0.3 to 0.4% for HbA1c, 5 to 10 mg/dL for fasting glucose and around five percentage points for CGM time-in-range, rather than against *p*-values alone. Table 2 provides a structured overview of the evidence across all classes, and a class-by-domain comparison appears later in this section.

### 5.1. Anthocyanins (Berries and Concentrates)

Anthocyanins provide the most coherent clinical signal among all polyphenol classes examined for T2D. Parallel and crossover RCTs using berry concentrates, freeze-dried powders or capsules, at doses of approximately 150 to 640 mg per day over 8 to 24 weeks, report consistent and modest improvements across the full glycaemic hierarchy, with fasting plasma glucose decreasing by approximately 5 to 10 mg/dL and HOMA-IR improving in parallel. For HbA1c, the most robust pooled estimate available is a reduction of 0.31 percentage points, reported by Mao and colleagues in adults with T2D [36]. Two features of that source constrain how it may be read, since the median intervention length was eight weeks, with eligible trials starting at four weeks, and none of the contributing trials verified metabolite exposure. We therefore report approximately 0.3 percentage points as the best current estimate for anthocyanins in T2D from unverified trials, an effect that is modest and sits at the lower bound of the 0.3 to 0.4 percentage point range generally treated as minimally important, and we note that the corresponding estimate under verified exposure is simply unknown, which is itself one of the central arguments of this review. Postprandial effects tend to be stronger and appear earlier, since meal-timed doses reduce the incremental AUC for glucose and insulin and, in the few trials using CGM, lower 24 h mean glucose and variability with gains in time-in-range [37]. OGTT-derived indices such as the Matsuda index move in the expected direction after eight to twelve weeks of dosing, although that particular evidence was obtained in adults with prediabetes or early untreated diabetes rather than in established T2D [38].

Trials with exposure verification detect early phase-II conjugates, mainly anthocyanin glucuronides and sulphates peaking at one to two hours, together with delayed microbial phenolic acid catabolites, consistent with biphasic pharmacology. These metabolite peaks track closely with surrogate cardiometabolic benefits, including reduced hsCRP and IL-6, improved flow-mediated dilation and tentative hepatic signals in participants with coexisting MASLD [39]. Null findings are mostly seen with durations under eight weeks, low doses, poorly matched comparators or an absence of metabolite verification.

Anthocyanin interventions were generally well tolerated in the trials reviewed, although the available follow-up is short and adverse events were not extracted systematically, so this should not be read as a formal safety assessment. Adherence exceeds 85 to 90% across formulations, and reported adverse events remain mild and transient, including bloating, dyspepsia and reversible stool discolouration, with no sustained hepatic or renal changes and no hypoglycaemia signals [40]. High-sugar berry juices should ideally be replaced with low-sugar beverages or standardised capsules in order to avoid glycaemic confounding, rare berry allergies remain possible, and the potassium content of these products makes high-volume fruit drinks inadvisable in advanced CKD.

**Figure 3:**
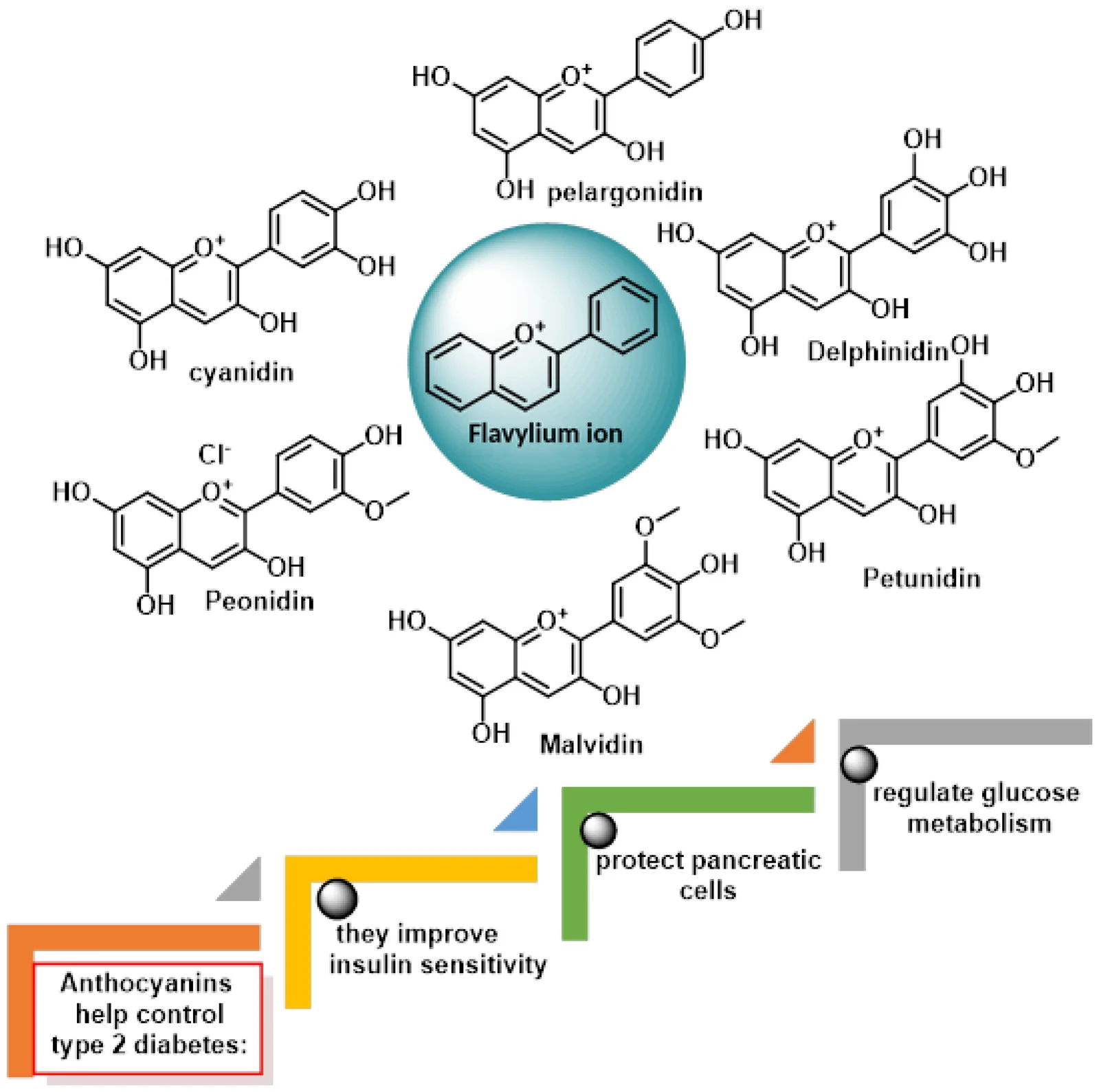
Basic aglycone structure of anthocyanins, composed of a flavylium ion (2-phenylbenzopyrylium), together with an overview of anthocyanin structure and its effects on diabetes.

Figure 3 depicts the flavylium ion core, known as 2-phenylbenzopyrylium, that all anthocyanins share, together with its relation to their observed effects in diabetes.

### 5.2. Stilbenes (Resveratrol)

The evidence base for resveratrol in T2D is mixed and requires a clear distinction between glycaemic and cardiometabolic outcomes. Across RCTs spanning 8 to 24 weeks with doses of 250 to 1000 mg per day of trans-resveratrol, fasting glucose and HOMA-IR show small and directionally favourable changes, whereas HbA1c benefits emerge inconsistently and generally require twelve weeks or more to appear [41]. Inflammatory and vascular readouts follow a considerably more coherent pattern, with reductions in hsCRP and in circulating lipid peroxides and rises in glutathione peroxidase and catalase activity among the more reproducible findings [42], while improvements in flow-mediated dilation are reported in a separate pooled analysis [43], although that pooled analysis graded its own certainty as low to very low and several markers did not change significantly. This distinction matters considerably in clinical terms, since vascular and inflammatory improvements could plausibly translate into downstream glycaemic benefit over longer exposure periods, which trials lasting only 8 to 12 weeks cannot capture, and the pooled vascular evidence cited here was obtained in adults with metabolic syndrome and related disorders rather than in established T2D [43].

**Figure 4:**
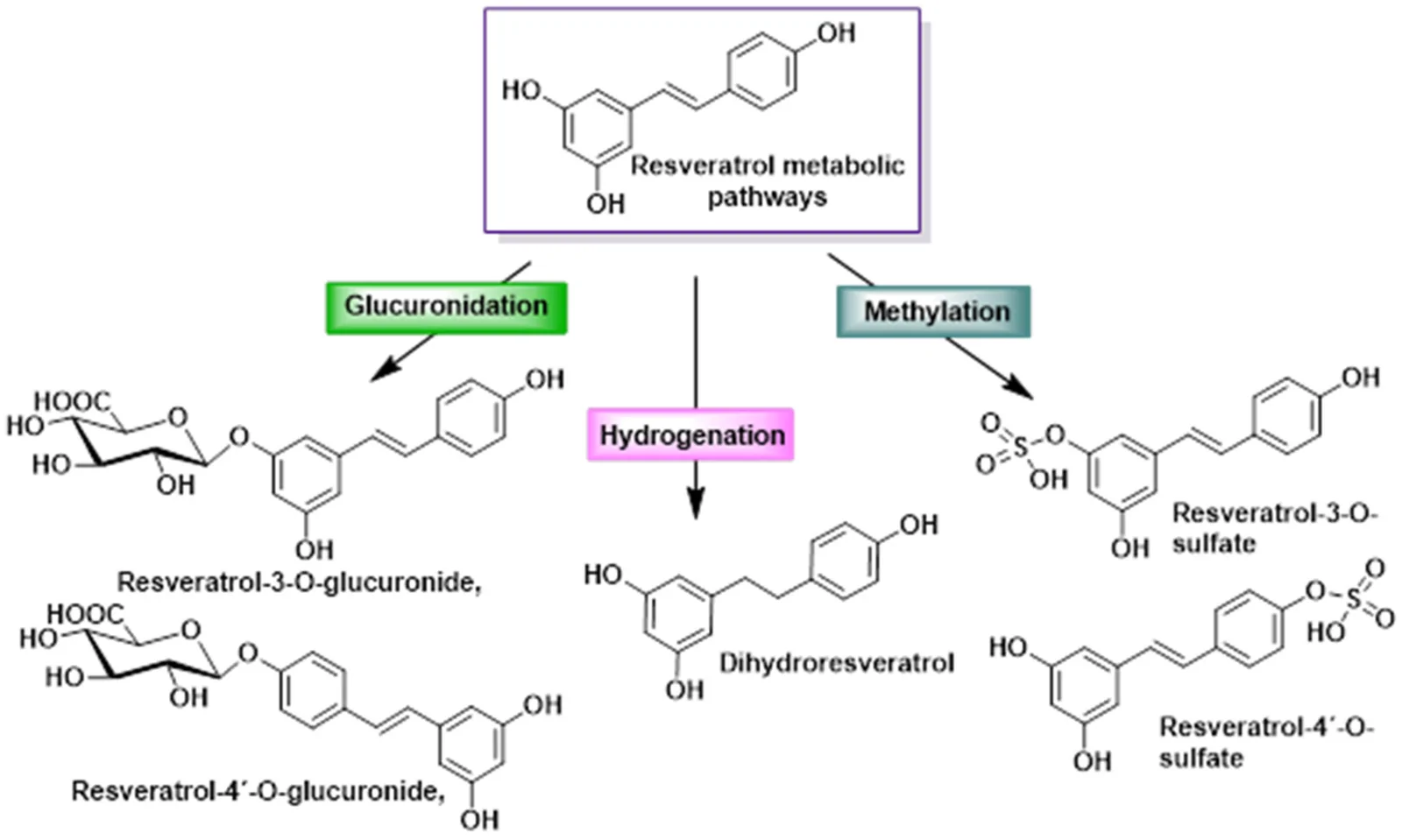
Formation of resveratrol metabolites through various UDP-glucuronosyltransferases (UGT) and cytosolic sulfotransferases (SULT) in humans.

Figure 4 outlines the conversion of resveratrol into its main circulating metabolites through the combined action of several UDP-glucuronosyltransferases and cytosolic sulfotransferases.

A key interpretive problem is that human systemic exposure to resveratrol is dominated by glucuronide and sulphate conjugates, together with microbiome-derived dihydroresveratrol, rather than by the aglycone itself [44], yet few trials verify these metabolites or sample the late window at all. Published meta-analyses generally report modest pooled glycaemic effects accompanied by high heterogeneity, and they share these same verification gaps [45]. Until metabolite-verified, meal-aligned trials lasting twelve weeks or longer are available, resveratrol is best positioned as a plausible cardiometabolic adjunct with variable and still inconsistent glycaemic effects.

### 5.3. Catechins and Green Tea (EGCG)

Green tea catechins, particularly EGCG, show promising but uneven evidence in T2D. RCTs using brewed tea or standardised extracts, at doses of approximately 300 to 800 mg per day of catechins over 8 to 24 weeks, consistently report small reductions in fasting glucose and HOMA-IR [46], while HbA1c improvements appear mainly in studies lasting twelve weeks or more, or in those including participants with poorer baseline control [47]. Postprandial outcomes improve reliably when dosing is meal-timed, consistent with both intestinal glucose transport modulation and the delayed appearance of γ-valerolactone catabolites during the late window [48].

**Figure 5:**
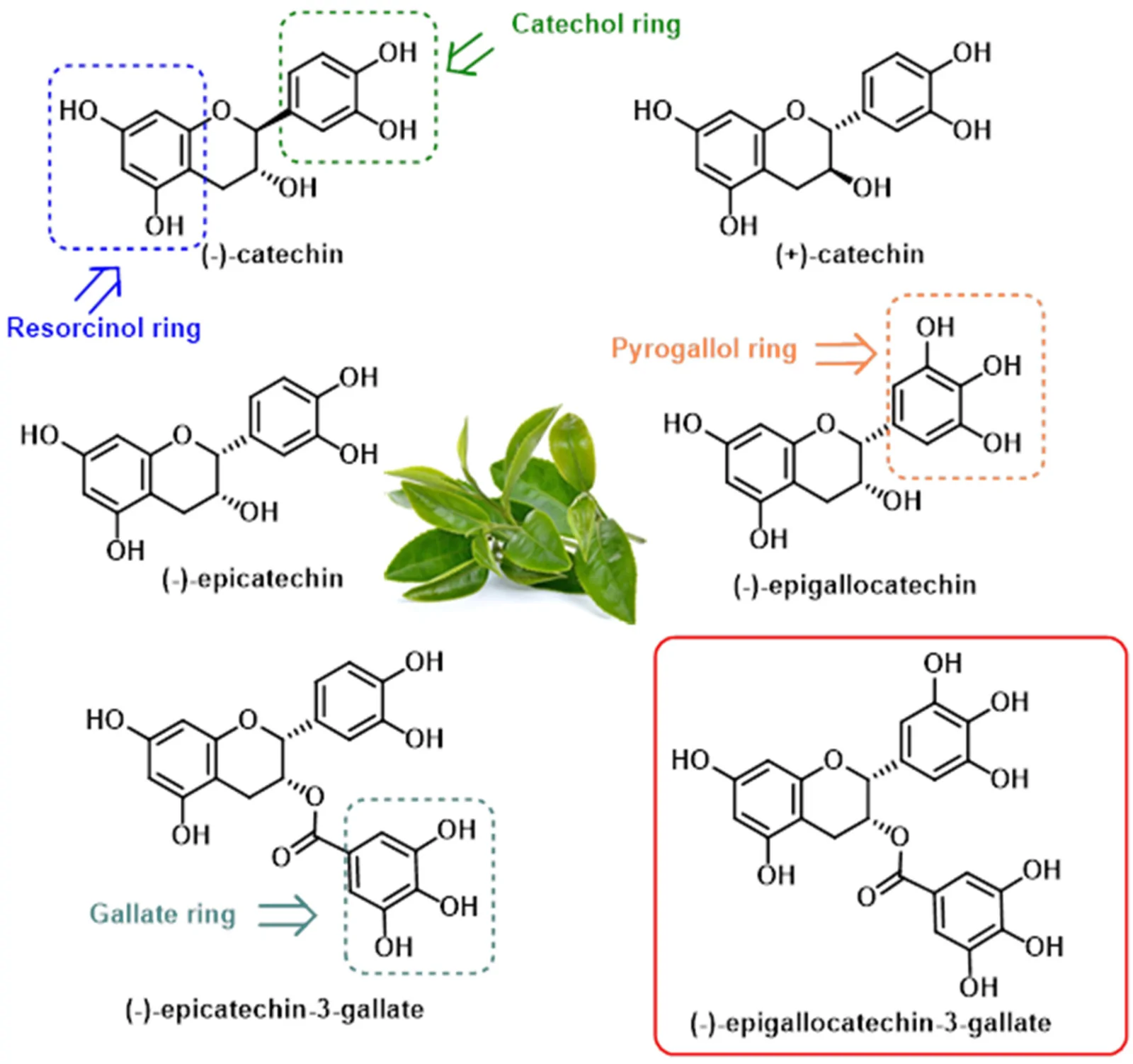
Chemical structure of green tea catechins.

Figure 5 shows the chemical structures of the catechins found in green tea.

Caffeine is a meaningful confounder throughout this literature, since it affects glycaemia and vascular readouts through sympathetic tone, energy expenditure, sleep quality and diuresis [49]. Isolating catechin-specific effects therefore requires decaffeinated extracts or at least caffeine-matched controls, together with explicit reporting of caffeine intake, and high-dose EGCG in the fasted state should generally be avoided given its altered tolerability and exposure kinetics. Standardised extracts provide considerably more reproducible dosing than brewed tea, whose catechin content varies with leaf grade, steeping time and water temperature. The overall signal is credible for fasting and postprandial control, although adequately powered, metabolite-verified, meal-aligned trials of twelve weeks or more are still limited.

### 5.4. Cocoa Flavanols

Cocoa flavanols show a notable divergence between trial scale and trial quality. Large prevention studies that use confectionery matrices, together with heterogeneous older populations and long intervals between assessments, have consistently failed to reduce incident T2D or to produce meaningful HbA1c changes [50]. This finding is unsurprising, since the matrix confounds phenolic attribution, metabolite verification remains absent, and disease incidence is a blunt endpoint for a nutrient signal of modest size.

Small, tightly controlled mechanistic studies using low-sugar beverages or standardised capsules, at doses of approximately 200 to 600 mg per day of total flavanols and 100 to 200 mg per day of (−)-epicatechin over 8 to 24 weeks, provide a different signal. These trials verify early exposure to conjugated (−)-epicatechin and to late γ-valerolactone catabolites, align sampling with the expected peaks, and consistently demonstrate improved flow-mediated dilation, modest blood pressure reductions and context-dependent improvements in HOMA-IR and hsCRP [51]. The underlying mechanism appears primarily vascular with metabolic crosstalk, rather than directly glycaemic, so the most practical next step is to target high-risk, insulin-resistant populations with low-sugar standardised preparations, to verify exposure across both metabolic windows, and to pair vascular readouts with CGM and twelve-week or longer HbA1c measurements.

### 5.5. Olive Phenolics (Hydroxytyrosol, Oleuropein, Extra-Virgin Olive Oil)

Extra-virgin olive oil, commonly abbreviated as EVOO, shows small but consistent improvements in postprandial glucose and insulin when it replaces refined oils at mixed meals, with gains in fasting indices and HOMA-IR emerging after twelve weeks or more [52], alongside clearer effects on endothelial function, hsCRP and hepatic markers in MASLD subgroups. The main problem of attribution is that the MUFA matrix of EVOO independently blunts glycaemic excursions, so isolating phenolic effects requires either phenolic-content-matched designs comparing high- with low-phenolic EVOO or trials using purified hydroxytyrosol at 5 to 25 mg per day or oleuropein at 50 to 100 mg per day. Few studies verify authentic exposure, and late-window sampling is almost universally absent from this literature.

**Figure 6:**
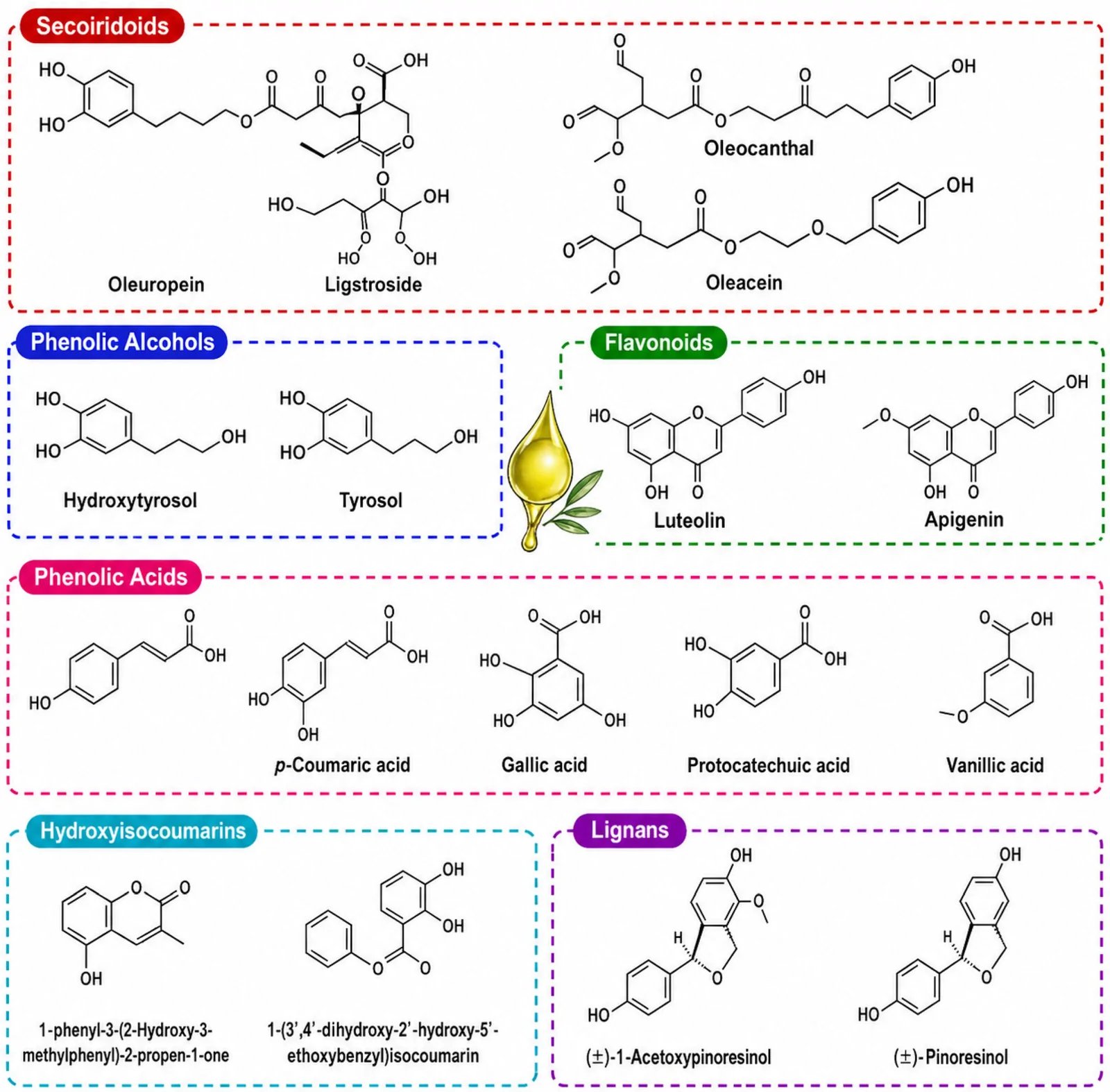
Representative diagram showing the main classes of polyphenols found in olive oil, including simple phenols, secoiridoids, phenolic acids, lignans, flavonoids and hydroxy-isochromans.

Figure 6 presents a representative overview of the main classes of polyphenols present in olive oil, illustrating the chemical diversity that underlies its health effects.

In lipid and inflammatory pathways, olive phenolics consistently lower triglycerides and oxidised LDL, improve LDL particle oxidisability and enhance HDL function, including cholesterol efflux capacity and paraoxonase activity, even when LDL-C concentrations themselves change relatively little [53]. Reductions in hsCRP, IL-6 and adhesion molecules track closely with endothelial improvements [54], and in MASLD subgroups transaminase declines run parallel to CRP reductions, pointing towards NF-κB dampening and NRF2 support as shared mechanisms [55]. Rigorous, metabolite-verified trials lasting twelve weeks or more, and capable of separating phenolic from MUFA effects, are the clearest next step for this particular class.

### 5.6. Other Classes: Isoflavones, Curcuminoids, Ellagitannins, and Procyanidins

Isoflavones, dosed at approximately 40 to 100 mg per day of genistein or daidzein over 8 to 24 weeks, show small and rather inconsistent effects on fasting glucose and HOMA-IR. The dominant determinant of response seems to be equol producer status, since individuals whose gut microbiota can convert daidzein into S-equol show systematically larger effects, yet the pooled isoflavone estimates available for T2D are not stratified by producer status and metabotype stratification is reported in only a small minority of trials [56]. Until producer status becomes routinely classified and balanced at randomisation, pooled estimates for isoflavones will remain systematically diluted by non-producers.

**Figure 7:**
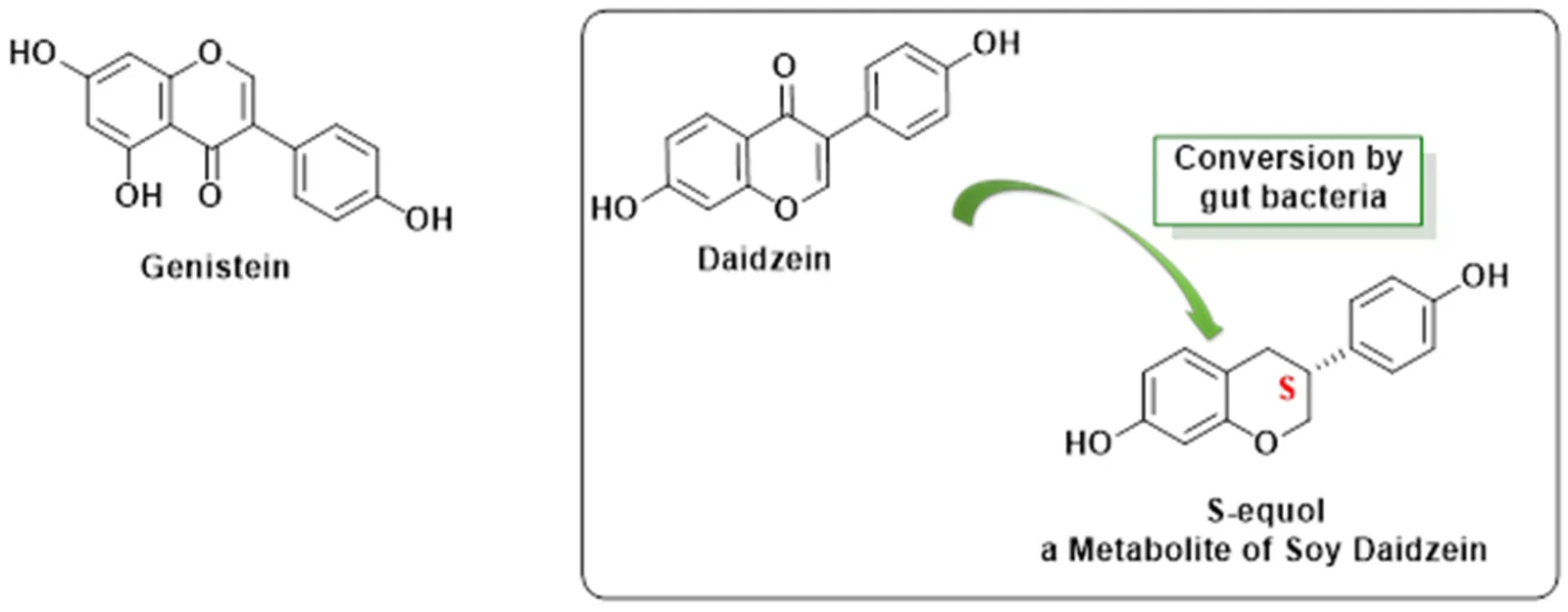
Chemical structure of genistein, daidzein and S-equol, together with the conversion of the isoflavone daidzein into S-equol by specific gut microbiota in the human digestive tract.

Figure 7 illustrates the chemical structures of genistein, daidzein and S-equol, together with the microbial conversion of daidzein into S-equol within the human digestive tract.

Curcuminoids, dosed at approximately 500 to 1500 mg per day and often combined with bioavailability enhancers, produce among the most reproducible anti-inflammatory findings in polyphenol nutrition research, with consistent reductions in hsCRP, TNF-α and IL-6 and occasional improvements in HOMA-IR [57]. HbA1c effects remain inconsistent, partly because poor solubility and rapid phase-II conjugation mean that nominal dose bears little relation to actual systemic exposure. Formulation heterogeneity is extreme in this field, since piperine, phospholipid complexes, nanoencapsulation and solid dispersions each alter bioavailability, CYP activity and P-gp transport in ways rarely characterised within published reports [58], so reliable T2D effect size estimates require verified plasma curcuminoid concentrations rather than nominal dose alone.

**Figure 8:**
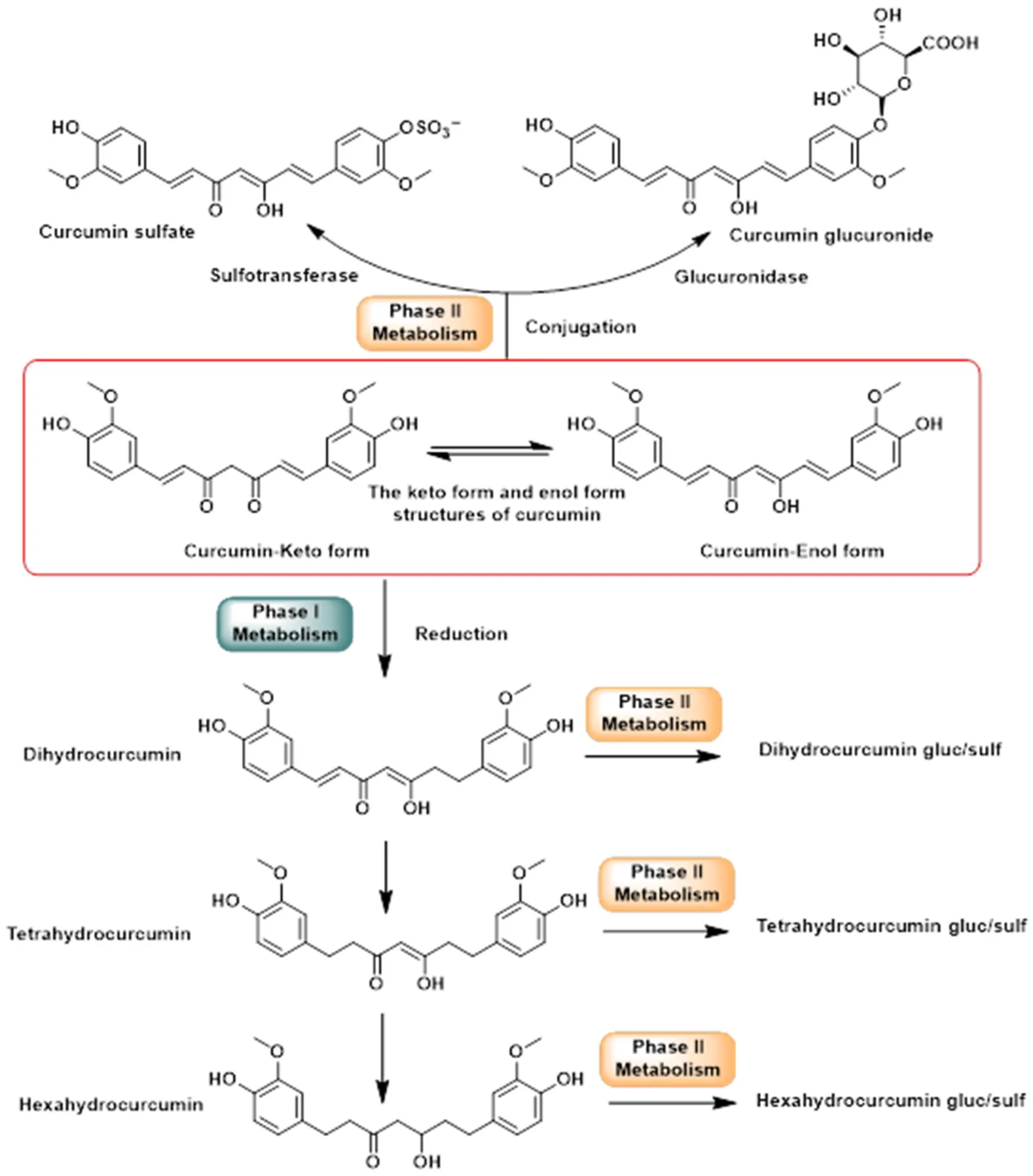
Metabolism of curcumin. The reduction pathway and the two main conjugation pathways, glucuronidation and sulfation, are shown. Curcumin is metabolised rapidly in the body and undergoes mainly phase I reduction metabolism followed by phase II conjugation metabolism.

Figure 8 depicts the metabolism of curcumin through the reduction pathway and through the two principal conjugation routes, namely glucuronidation and sulfation.

**Figure 9:**
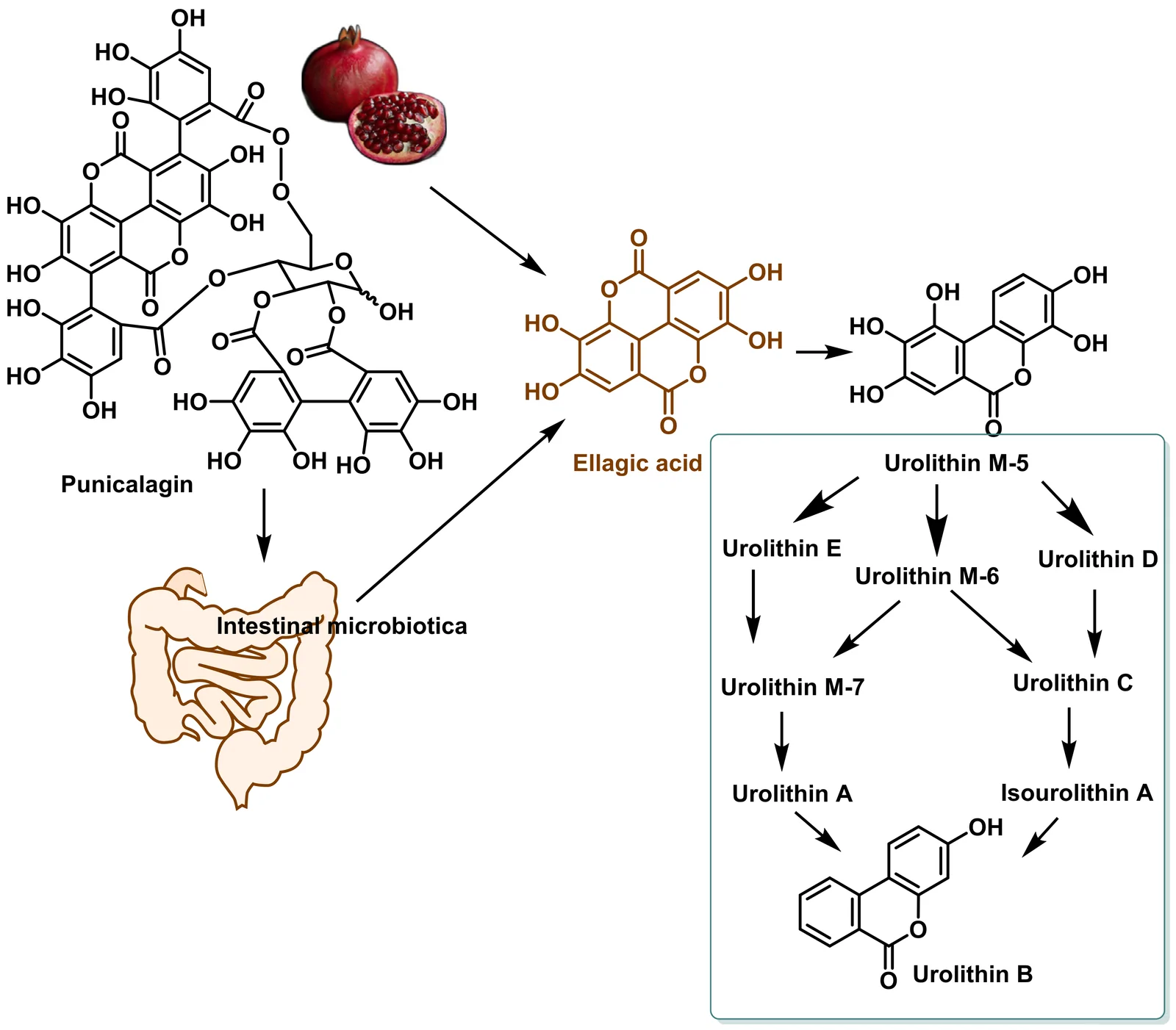
Biodegradation of ellagitannins and ellagic acid into urolithins through the action of the gut microbiota. Pomegranate has been reported to reduce fasting glucose, fasting insulin and HOMA-IR in adults, particularly in patients with type 2 diabetes.

Ellagitannins, found in pomegranate, walnuts and certain berries, remain mechanistically attractive because of their urolithin catabolites, although direct glycaemic RCTs in T2D are few and mostly show modest or non-significant effects [59]. The methodological gap is straightforward, since pooling urolithin non-producers, who represent 25 to 40% of adults, together with producers systematically attenuates any real signal, so pre-screening and stratified randomisation by urolithin metabotype should be considered wherever genuinely interpretable trials are intended. Procyanidins, found in apple skin, grape seeds and certain berries, show encouraging short-term effects on postprandial glucose and endothelial function, although adequately powered trials of twelve weeks or more, using CGM and HbA1c as primary endpoints, remain lacking [60]. Safety at nutritional doses is reassuring across these classes. Figure 9 illustrates the biodegradation of ellagitannins and ellagic acid into urolithins through the action of the gut microbiota.

### 5.7. Whole-Food and Dietary-Pattern Interventions

Polyphenol-rich dietary patterns, including Mediterranean, Nordic and other plant-forward diets, consistently improve postprandial control and cardiometabolic intermediates in T2D, with HbA1c benefits emerging after twelve weeks or more [61], particularly when the intervention also includes weight stabilisation or modest weight loss. Attributing these effects to polyphenols specifically remains inherently difficult, because fibre, unsaturated fats and a lower glycaemic load all operate in parallel within these diets. The most informative designs therefore standardise phenolic content, comparing, for example, high with low phenolic EVOO, align dosing with meals and earlier eating windows, and pair clinical endpoints such as HbA1c, CGM and hepatic fat with targeted metabolomics. These patterns remain safe, acceptable and clinically useful overall, and metabolite-verified, energy-matched trials will help clarify the phenolic-specific share of this benefit.

**Figure 10:**
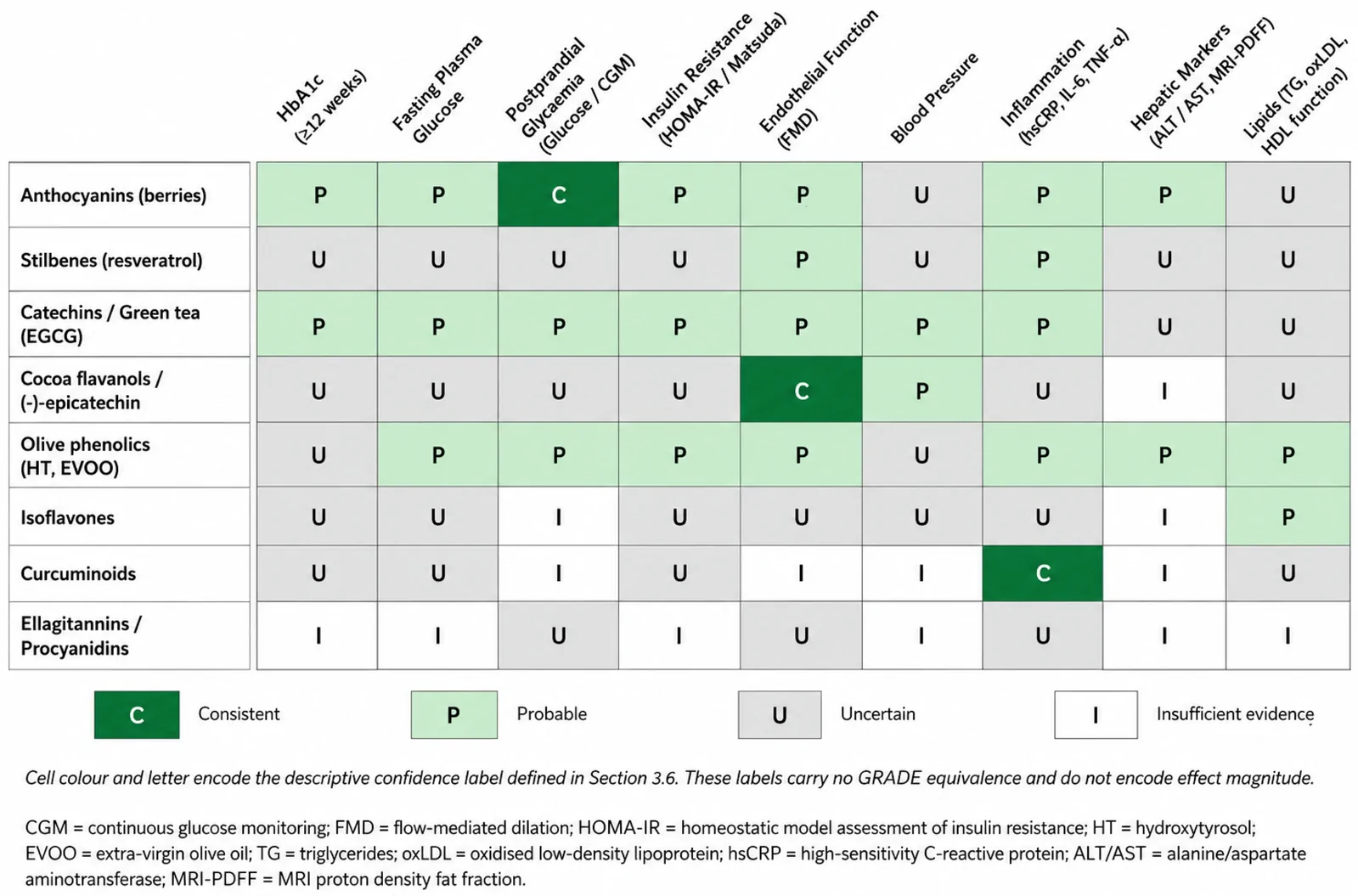
Summary of glycaemic and cardiometabolic effects by polyphenol class in adults with T2D. Conceptual summary matrix prepared by the authors. Cell colour and the letter shown in each cell encode the descriptive confidence label defined in Section 3.6, carry no GRADE equivalence and do not encode effect magnitude. Dark green (C) = consistent, meaning the direction of effect is reproduced across several independent randomised trials of adequate duration. Light green (P) = probable, meaning it is reproduced in some trials but not others, or rests mainly on secondary endpoints. Grey (U) = uncertain, meaning the evidence is inconsistent or too limited for a directional statement. White (I) = insufficient evidence, meaning no eligible randomised trial in adults with T2D was identified for that outcome domain.

Figure 10 provides an at-a-glance summary of the glycaemic and cardiometabolic evidence across the eight polyphenol classes reviewed here, organised by outcome domain and labelled using the descriptive confidence terminology defined in Section 3.6 rather than GRADE. The only consistent glycaemic benefit is that of anthocyanins on postprandial control, whereas vascular and inflammatory signals are distributed more broadly across classes, with cocoa flavanols and resveratrol showing their strongest evidence precisely within these non-glycaemic domains. The widespread grey and white cells reflect the systematic absence of metabolite verification and adequate trial duration within the existing literature, rather than clear evidence of biological inactivity, and they highlight where future well-designed trials are most urgently needed.

## 6. Mechanistic Signals Relevant to Human T2D

Human polyphenol trials show reproducible signals across six axes, namely insulin sensitivity and glucose uptake, β-cell efficiency and incretin function, hepatic steatosis and lipotoxicity, systemic inflammation and oxidative stress, endothelial and microvascular function, and mitochondrial quality control. Early host conjugates act primarily at the intestinal-hepatic interface within the first four hours, whereas microbiome catabolites arriving during the late window reach extra-intestinal tissues, and timing, matrix and metabotype together determine which of these axes respond and by how much.

### 6.1. Insulin Sensitivity and Glucose Uptake

Human trials consistently show small improvements in fasting insulin, HOMA-IR and OGTT-derived indices after 8 to 12 weeks, pointing towards a peripheral mechanism. Late-window microbiome catabolites circulating as conjugates are the most plausible extra-intestinal drivers, since they activate AMPK and nutrient sensing pathways, enhance fatty acid oxidation and alleviate lipotoxicity-mediated IRS-1 serine phosphorylation [62], while in adipose tissue they attenuate NF-κB activity and favour adiponectin secretion [63]. A complementary mechanism involves microvascular recruitment, whereby improved endothelial nitric oxide bioavailability boosts nutritive blood flow to skeletal muscle and facilitates glucose disposal, which is visible on CGM as attenuated peaks and increased time-in-range [64].

### 6.2. β-Cell Function and Incretin Axis

Polyphenol interventions frequently show lower postprandial insulin exposure at comparable glycaemia, implying less secretory demand per unit of glycaemic load. Disposition index surrogates occasionally improve in oral tests, whereas fasting *C*-peptide and HOMA-B change relatively little [65]. GLP-1 rises transiently with meal-aligned dosing in small studies and DPP-4 activity declines modestly in some trials [66], although both signals remain inconsistent and require metabolite verification. A simpler mechanism, namely intestinal glucose transport modulation producing flatter excursions, reduces β-cell stimulus independently of any incretin effect [67], so these findings together suggest improved postprandial efficiency rather than durable changes in basal insulin secretion.

### 6.3. Hepatic Outcomes: Steatosis, Transaminases, MRI-PDFF

Where measured, ALT and AST tend to decrease and MRI-PDFF occasionally shows modest fat reduction, most clearly when interventions last twelve weeks or more with controlled energy intake and minimal weight change [68]. These effects appear amplified in participants with MASLD, consistent with early conjugates acting at the intestinal-hepatic interface and late catabolites dampening de novo lipogenesis [69]. EVOO trials must separate phenolic from MUFA effects and cocoa studies must control for sugar and fat matrices, while metabolite verification remains infrequent. Hepatic evidence therefore looks cautiously positive and warrants metabolite-verified, energy-matched designs of twelve weeks or more with prespecified hepatic endpoints.

### 6.4. Inflammation and Oxidative Stress

The clearest and most consistent non-glycaemic polyphenol signal in T2D is a small anti-inflammatory shift. Modest reductions in hsCRP and, less uniformly, in IL-6 and TNF-α accompany well-controlled interventions, with effects largest when exposure is verified and duration reaches twelve weeks or more. Vascular readouts often move in parallel, suggesting a coordinated dampening of endothelial activation rather than isolated cytokine changes [70]. For oxidative stress, assay quality is decisive, since studies using validated markers such as F_2_-isoprostanes and oxidised LDL show small improvements with meal-timed sustained dosing, whereas TBARS and generic total antioxidant capacity assays remain noisy and should not drive inference [71].

### 6.5. Endothelial Function and Microvascular Health

Polyphenol interventions consistently improve endothelial function, showing modest gains in flow-mediated dilation and, in some studies, reduced augmentation index or pulse wave velocity [72], while microvascular readouts improve in parallel when dosing is meal-timed. Increased nitric oxide bioavailability through eNOS activation, reduced endothelin-1 tone and dampened vascular inflammation together represent the main mechanistic drivers [73], and improved microvascular recruitment enhances insulin and glucose delivery to skeletal muscle, aligning with HOMA-IR improvements and gains in CGM time-in-range, as observed in a trial conducted in insulin-resistant adults without diabetes [74]. Effects appear larger in participants with poorer baseline vascular function and with confirmed metabolite exposure.

### 6.6. Mitochondrial Function and Mitophagy

Human evidence linking polyphenols to mitochondrial quality control remains suggestive and preliminary. Small trials report lower circulating acylcarnitines, modest rises in resting energy expenditure and higher ex vivo respiratory capacity after sustained intake [75], and early-phase urolithin A studies show transcriptional activation of TFEB programmes together with small functional gains [76]. Data on γ-valerolactones remain more limited, although mechanistically aligned through AMPK activation [77]. The two-window model provides a coherent explanation, since early conjugates dampen oxidative and inflammatory noise while late microbiome metabolites nudge the AMPK-SIRT1-PGC-1α axis and facilitate mitochondrial turnover [78]. These effects depend on dose, on duration of eight to twelve weeks or more, and on verified exposure.

## 7. Sources of Heterogeneity and How to Read the Literature

A central proposition of this review is that at least part of the heterogeneity observed in polyphenol trials reflects structured biology rather than random variability, although this remains undemonstrated because the necessary treatment-by-metabolite and treatment-by-metabotype interaction analyses have not been reported. The main drivers include matrix, dose and formulation, timing relative to meals and circadian phase, metabolite verification status, person-level metabotype, transporter genetics, background diet and concomitant medications. Pooling across all of them produces wide confidence intervals and inconsistent estimates that are better treated as signals worth dissecting than as failures needing resolution [79].

A practical checklist is genuinely helpful when reading this literature. Readers should ask whether comparators are matched for energy and macronutrient composition rather than for sugar alone, whether duration reaches twelve weeks or more for HbA1c, whether analyses are intention-to-treat and consistent with the registry prespecification, whether endpoints are paired with metabolite verification, and whether small effects are judged against minimally important differences rather than *p*-values. Trials that pass this checklist deserve the greatest weight in any synthesis.

### 7.1. Matrix, Dose, Formulation, and Co-Nutrients

The phenolic dose cannot be separated from the matrix that carries it into the body. High-sugar juices raise glycaemic load and confound attribution, whereas low-sugar beverages and standardised capsules are considerably more interpretable. Fat and emulsifiers boost the early conjugate window; viscous fibre instead shifts substrate towards the late microbiome window, and protein can bind phenolics and reduce the bioavailable fraction. Standardised extracts dose more reproducibly than brewed tea, sustained-release formulations can retime exposure, and decaffeinated extracts reduce caffeine confounding [80]. Dose, matrix, timing and metabolite verification should serve as primary intervention descriptors in every published report.

### 7.2. Metabolite Verification: What to Measure, When, and How

Verification of authentic exposure should be standard practice, rather than an optional extra in trial design. Both windows must be sampled, covering 0.5 to 4 h post dose for phase-II conjugates and 6 to 24 h for microbiome catabolites, in plasma together with spot or 24 h urine and including a fasting trough. Quantification requires targeted LC-MS/MS with stable-isotope internal standards and reporting of LLOQ, recovery and stability, while meal content, clock time and co-ingested caffeine or medications should be documented and the analyte panel preregistered. Table 3 gives a practical reference panel for each polyphenol class, and trials lacking verification should be clearly flagged in any sensitivity analyses.

### 7.3. Metabotypes: Responders and Non-Responders

Metabotype is defined by an individual’s capacity to generate microbiome catabolites following a standardised polyphenol challenge. A pre-screening panel comprising urolithin A-3-glucuronide, a representative γ-valerolactone glucuronide and S-equol, measured in plasma or urine at 8 to 12 h plus a fasting trough, classifies producer status before randomisation [81]. Stratified randomisation with prespecified treatment-by-metabotype interaction tests is recommended in order to detect subgroup-specific effects reliably, while intention-to-treat remains the primary analysis, and for non-producers direct metabolite supplementation or alternative polyphenol classes are logical substitutes, although the supporting exposure data come from healthy adults rather than from people with T2D [82].

**Figure 11:**
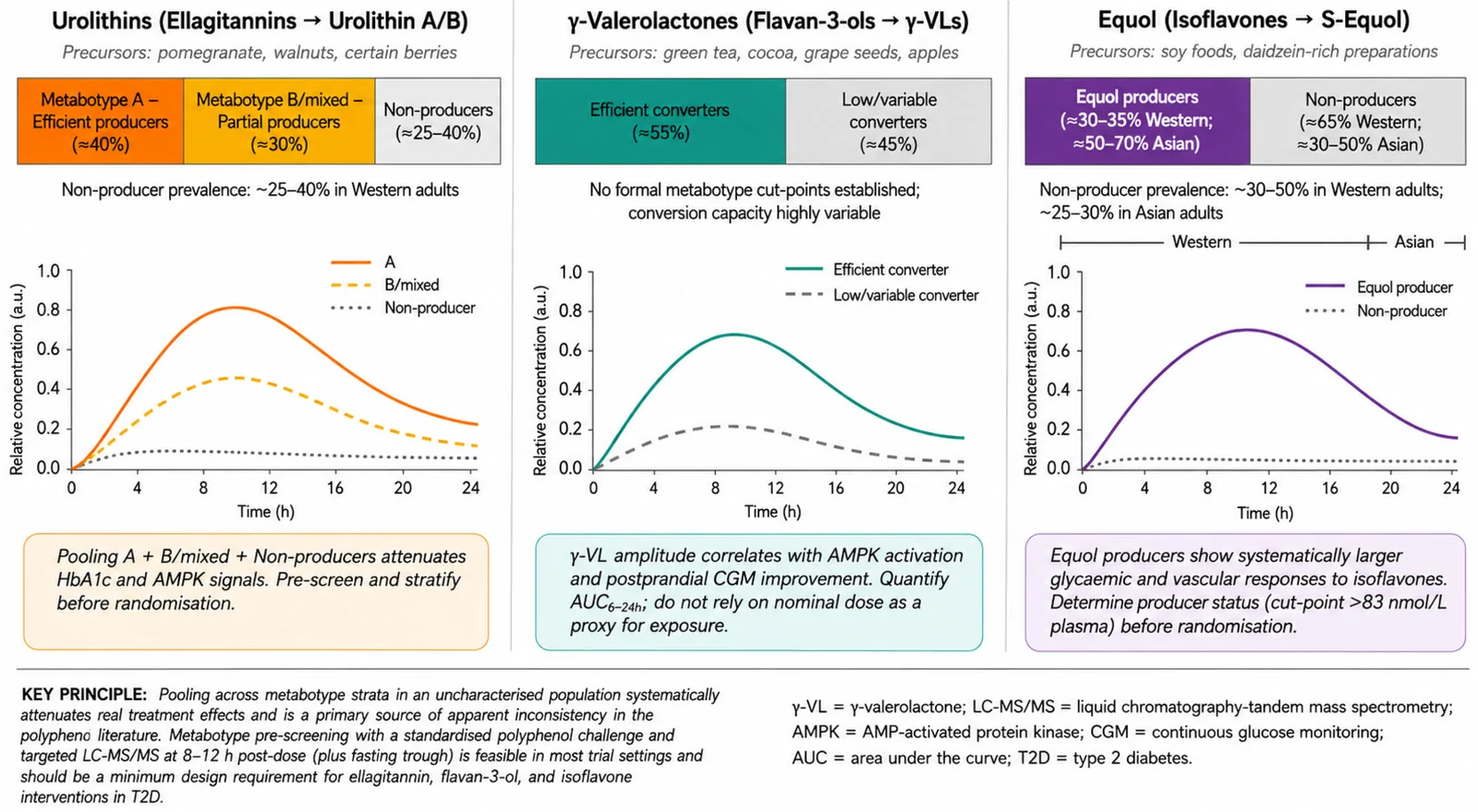
Conceptual diagram, not drawn to scale. Metabotype-driven variability in polyphenol response. Individual capacity to generate urolithins, γ-valerolactones and equol determines late-window metabolite profiles, and non-producer prevalence is approximately 25–40% for urolithins and 30–50% for equol in Western populations. Pooling across metabotypes would be expected to attenuate any real effect, although this has not yet been demonstrated in trials with glycaemic endpoints.

Figure 11 is a conceptual, not-to-scale map of three principal axes of microbiome-driven metabotype variability that shape late-window exposure, and the distributions and concentration-time curves shown are schematic rather than derived from any dataset, model fit or measured population sample. It illustrates why pooling across uncharacterised populations would be expected to attenuate a treatment effect if one exists, since non-producers, who represent up to 40% of Western adults for urolithins and up to 50% for equol, contribute little or no late-window exposure regardless of dose. Whether this accounts for the heterogeneity seen in the T2D literature has not been tested, because no glycaemic trial has reported a prespecified treatment-by-metabotype interaction analysis.

### 7.4. Concomitant Therapies

Background diabetes therapy shapes both baseline control and the detectable size of any nutritional signal observed. Metformin modulates AMPK and the gut microbiome and may amplify late-window catabolite generation [83], incretin-based agents blunt postprandial excursions and slow gastric emptying, SGLT2 inhibitors lower fasting and postprandial glucose while promoting weight loss, and insulin together with thiazolidinediones can mask small nutritional effects [84]. Stable regimens should therefore be required for eight to twelve weeks or more before randomisation, therapy classes balanced across arms, subgroup analyses by drug class prespecified, and all therapy changes documented together with sensitivity analyses censoring post-change data.

### 7.5. Adherence, Diet, and Lifestyle

Adherence determines whether the nominal dose becomes real exposure within the body. Capsule counts and electronic diaries should be supplemented by biochemical verification within a representative subset of participants, using validated metabolite biomarkers of intake [85]. Background diet should be standardised through menu cycles or energy-matched meal kits, extraneous polyphenol intake, caffeine and alcohol should be controlled around the sampling windows, weight stability should be targeted unless weight loss is a prespecified endpoint, and both intention-to-treat and adherence-sensitive analyses should be reported.

### 7.6. Study Design Pitfalls

Several recurring design problems can blunt real signals across this literature. Acute meal tests are overinterpreted as evidence of chronic efficacy when trials of twelve weeks or more are required to assess HbA1c, underpowered studies chase significance across secondary endpoints that were never prespecified, comparators are energy-matched but not macronutrient-matched, and metabolite verification is often absent altogether. Single-time point sampling, inadequate washout in crossover designs, per-protocol drift away from intention-to-treat, and non-validated oxidative stress assays such as TBARS and generic total antioxidant capacity compound these issues further [86]. Preregistered, clinically anchored endpoints with twelve weeks or more of follow-up are the foundation of interpretable trials in this field.

## 8. Practical Implications

### 8.1. What Clinicians Can Reasonably Expect

Clinicians should expect modest, meal-contingent benefits, rather than drug-level glycaemic reductions from these interventions. The most reliable gains appear postprandially, with flatter glycaemic excursions and small improvements in fasting glucose and HOMA-IR. For HbA1c, the pooled estimate available for the best-supported class, anthocyanins, is a reduction of about 0.3% over a median of eight weeks in trials without metabolite verification, which is modest and lies at the lower bound of the minimally important difference of 0.3 to 0.4%, and this should be treated as the realistic order of magnitude rather than as a floor that longer or verified exposure might exceed, since no such estimate exists. Effects tend to be larger with poorer baseline control, prominent postprandial hyperglycaemia, coexisting MASLD and suboptimal vascular function. Anthocyanins offer the steadiest glycaemic signal, high-phenolic EVOO and green tea catechins are credible adjuncts when meal-timed and formulated without excess sugar or caffeine, and cocoa flavanols and resveratrol offer more consistent vascular and anti-inflammatory benefits. Most interventions were well tolerated at nutritional doses, although concentrated extracts, high-dose preparations and bioavailability enhancers require the cautions set out in Section 8.2, and CGM remains the most sensitive tool for detecting postprandial benefit [87].

### 8.2. Safety and Drug–Nutrient Interactions

Most adverse events reported across these trials are mild and transient gastrointestinal complaints, and high-sugar juices should generally be replaced with low-sugar beverages or standardised capsules. High-dose fasted EGCG carries a rare risk of hepatic enzyme elevation, so meal-timed and decaffeinated preparations are safer in practice [88]. Resveratrol and concentrated phenolics carry theoretical antiplatelet risks together with CYP3A4, CYP2C9, P-gp and BCRP interaction risks, so caution is warranted with warfarin and with strong substrates of these systems [89], and curcuminoids combined with piperine can raise the systemic exposure of co-administered medications [90]. PPIs and antibiotics attenuate late-window catabolite generation, iron supplements taken near tea or cocoa reduce mineral absorption [91], patients with advanced CKD should avoid high-volume fruit drinks because of their potassium load, and data covering pregnancy and lactation are insufficient at present. Table 4 summarises these signals by class, mechanism, clinical relevance and practical recommendation, and it requires an explicit caveat. We did not conduct a systematic search for drug–nutrient interactions, and the entries combine evidence of very different weight, from human pharmacokinetic studies and isolated case reports through to in vitro inhibition data and mechanistic inference. Table 4 should therefore be read as a cautionary listing of theoretical and preclinical interaction signals that merit attention rather than as a validated clinical interaction guide. The type of evidence is now stated for each row, and no entry should override the prescribing information for the drug concerned.

### 8.3. Patient Profiles Most Likely to Benefit

Benefits are most likely in adults with prominent postprandial hyperglycaemia, moderate to poor baseline control, and coexisting MASLD or vascular dysfunction, where endothelial and inflammatory improvements may translate into glycaemic gains [92]. Low habitual polyphenol intake and earlier, more consistent eating windows amplify these effects further. Urolithin A and γ-valerolactone producers may gain more from berry, cocoa and green tea strategies, whereas non-producer status or recent antibiotic or PPI use could blunt the late-window response unless direct metabolite formulations are used [93]. Patients already on potent GLP-1RA or SGLT2i therapy with near-target HbA1c are least likely to benefit further, and CGM together with targeted metabolite checks are the most informative personalisation tools currently available.

### 8.4. Separating Current Clinical Guidance from Research Recommendations

This review moves between two registers that are easily conflated, namely what a clinician may reasonably do with a patient today and what an investigator should build into the next generation of trials. Table 5 separates them explicitly, so that the design recommendations developed in Section 9 are not read as clinical guidance. Nothing in the research column is ready for routine care, and nothing in the clinical column substitutes for established glucose-lowering therapy.

## 9. Recommendations for Future Trials

**Figure 12:**
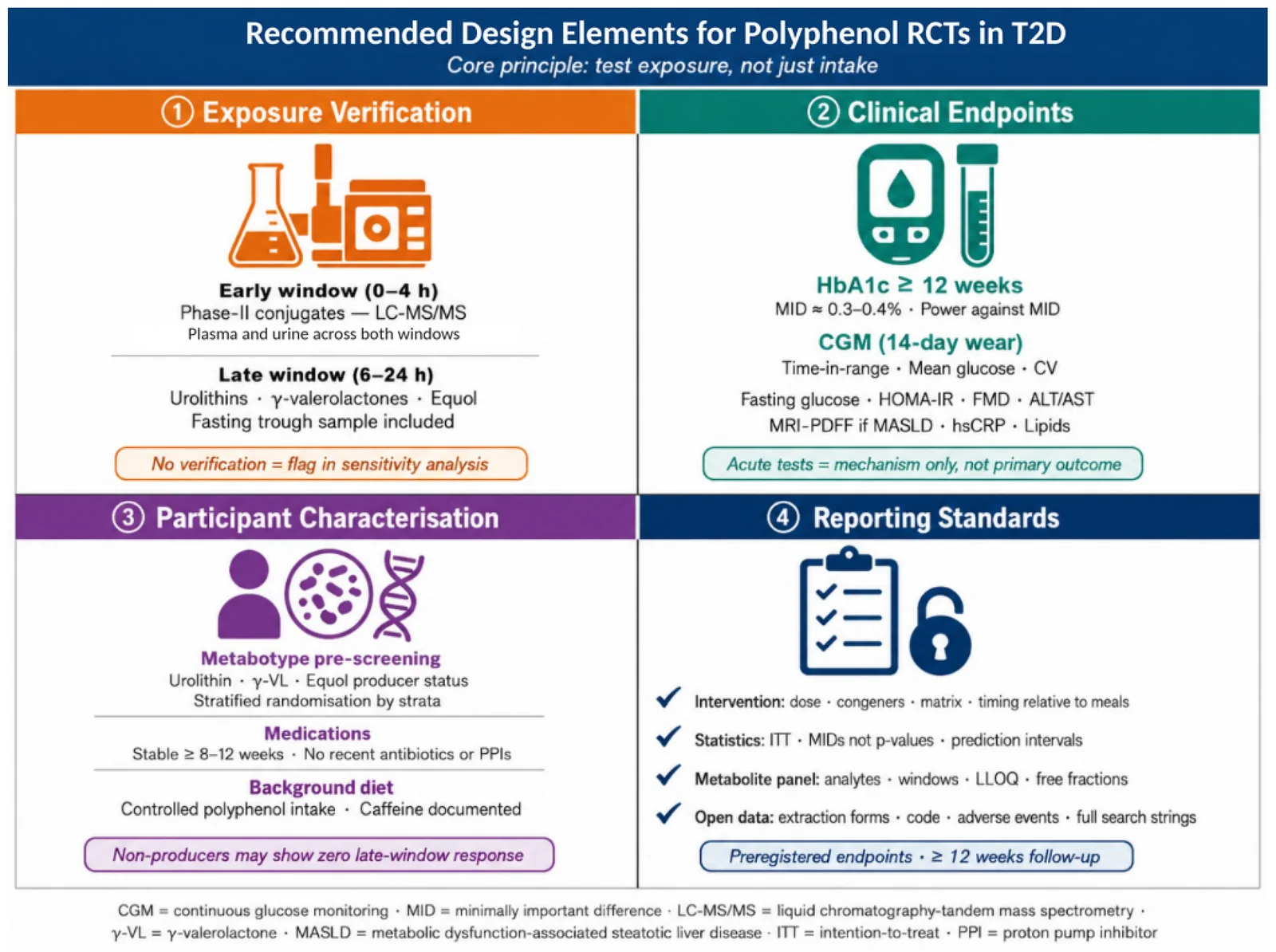
Recommended design elements for polyphenol RCTs in T2D, organised by domain: (1) exposure verification (targeted LC-MS/MS, both windows); (2) clinical endpoints (HbA1c ≥ 12 weeks + CGM primary); (3) participant characterisation (metabotype, medications, background diet); (4) reporting standards (transparent methods, open data).

Figure 12 condenses these design recommendations into a single-page reference across four operational domains, namely exposure verification across both metabolite windows, clinical endpoints anchored in HbA1c with CGM as the key secondary measure, participant characterisation by metabotype and background medication, and reporting standards. Together they represent the design elements that we recommend for a methodologically credible polyphenol RCT in T2D.

### 9.1. Core Design Principles

Investigators should pre-register their protocols and power them for minimally important differences, meaning approximately 0.3 to 0.4% for HbA1c and 5 to 10 mg/dL for fasting plasma glucose, while requiring stable background therapy for eight to twelve weeks or more before randomisation [94]. Energy and macronutrient-matched controls should be used throughout, with low sugar content and caffeine matching for tea-based interventions, meal-timed dosing specified in the protocol, and participants pre-screened and stratified by metabotype wherever this proves feasible. Concomitant therapies, recent antibiotic or PPI use, caffeine intake and background diet should all be documented, and genotyping of ABCG2 and ABCB1 can be informative wherever it is scientifically justified.

### 9.2. Metabolite Verification

Authentic exposure should be verified using targeted LC-MS/MS across both time windows, covering 0 to 4 h for phase-II conjugates and 6 to 24 h for urolithins, γ-valerolactones and phenyl or hippurate derivatives, in plasma alongside spot or 24 h urine and including a fasting trough. The core analyte panel and sampling windows are those set out in Table 3. LLOQ, recovery and stability should be reported using stable-isotope internal standards and blinded quality controls, and exposure indices such as AUC across each window, Cmax and tmax should be linked to pharmacodynamic readouts through prespecified exposure-response models.

### 9.3. Clinical Endpoints

HbA1c, assessed at twelve weeks or more, should serve as the primary endpoint for chronic efficacy, interpreted against a minimally important difference of approximately 0.3 to 0.4%. CGM is the key secondary endpoint, with fourteen days of wear per assessment and with time-in-range, mean glucose, coefficient of variation and postprandial time-above-range prespecified in advance [95]. OGTT-based mechanistic indices should be included under standardised conditions, hepatic endpoints added where MASLD is prevalent, and glycaemic outcomes paired with validated inflammatory and redox markers together with flow-mediated dilation.

### 9.4. Metabotype Stratification and Microbiome Characterisation

Participants should be classified before randomisation using a standardised polyphenol challenge with targeted metabolomics at 8 to 12 h plus a fasting trough, defining urolithin-A-producer, γ-valerolactone-converter, equol-producer and non-producer strata for block randomisation, with treatment-by-metabotype interaction tests prespecified from the outset. Stool should be collected at baseline and at the end of the trial for shotgun metagenomics targeting the relevant catabolism pathways [96], and by-metabotype effect sizes reported with prediction intervals alongside sensitivity analyses excluding participants who switch metabotype.

### 9.5. Chrononutrition and Formulation

We propose as a research hypothesis, and not as a clinical recommendation, that dosing aligned with the first main meal within a stable, earlier eating window may align the early conjugate peak with morning insulin sensitivity while allowing the microbiome wave to peak during a biologically active period. As already noted in Section 4.4, this rests on mechanistic rationale and indirect chrononutrition evidence rather than on polyphenol-specific factorial timing trials, so studies designed to test it should include an early versus standard timing arm together with a short run-in to standardise mealtimes. Until such data become available, timing guidance should be communicated as a biologically motivated optimisation rather than as an established intervention with a quantified effect size.

Formulation can be used to tune the exposure profile, using immediate-release or emulsified products for early-window amplification, sustained-release formulations to retime delivery, low-sugar vehicles to avoid glycaemic confounding, and decaffeinated extracts wherever caffeine sensitivity is a genuine concern. Pharmacokinetic sampling should be embedded at both time windows alongside CGM, and adherence to timing should be reported explicitly, with any misaligned dosing triggering prespecified sensitivity analyses.

### 9.6. Minimal Reporting Checklist

Every polyphenol RCT report should include ten elements, namely a power calculation against a minimally important difference, a clear account of randomisation, blinding and the intention-to-treat plan, full intervention details covering polyphenol class, daily dose, matrix, formulation, timing and duration, comparator details confirming energy and macronutrient matching, metabolite verification across both windows with LLOQ, recovery and stability, adherence data combining diaries with biochemical checks, background information on diet, weight, medications and recent antibiotic or PPI use, outcomes covering HbA1c at twelve weeks or more together with CGM, fasting and postprandial indices and validated marker panels, harms and laboratory safety data, and finally data and code availability together with the exact search strings used.

## 10. Limitations of the Evidence and of This Review

The clinical literature has several structural weaknesses that constrain inference. Most trials remain small, short and underpowered for detecting HbA1c differences at the minimally important level, and they rely on fasting or single-point postprandial surrogates rather than on CGM [97]. Matrix and formulation frequently confound attribution, since sugar in juices, fat in chocolate and MUFA in EVOO all require matched comparators that few studies actually use. Even otherwise sound trial designs suffer from the near-universal absence of metabolite verification, because distinguishing true non-response from mere non-exposure requires sampling both windows. Variable sampling windows, limited free-fraction reporting and inconsistent handling of background therapies that can mask or mimic small nutritional effects all compound this underlying problem further.

Assay quality for inflammation and oxidative stress remains a persistent weak link throughout this literature. F_2_-isoprostanes and oxLDL tend to produce genuinely interpretable results, whereas TBARS and total antioxidant capacity assays instead produce considerable noise, and hepatic outcomes look promising although the available evidence is still sparse. Large cocoa flavanol trials illustrate this blunt-endpoint problem clearly, since disease incidence provides a poor readout for a modest nutrient signal when dose, matrix and verification all remain suboptimal [98]. Metabotype stratification determined a priori is arguably the most important design feature for precision polyphenol nutrition, yet we identified it in only a small minority of the trials we examined [99], which is offered as an impression drawn from the studies reviewed rather than as a counted figure.

This review also has limitations. Our conclusions depend heavily on what individual trials have reported and on our ability to harmonise disparate outcomes and units across studies. Some polyphenol classes rest on a narrow evidence base, which requires placing undue weight on secondary endpoints or on small mechanistic studies, and our metabolite-centric emphasis may down-weight exposure-unverified trials even when real exposure genuinely occurred but simply went unmeasured. We did not conduct individual patient-data analyses, a choice which precludes formal treatment-by-metabotype or treatment-by-timing interaction tests; publication and language biases remain entirely possible despite our broad search coverage, while durable effects on micro and macrovascular complications remain largely untested at present.

A specific inferential limitation concerns chrononutrition. Our timing recommendations for morning-aligned, meal-timed dosing are grounded in mechanistic plausibility and general chrononutrition evidence rather than in dedicated polyphenol timing trials [100], and the effect-size estimates cited throughout do not include any independently quantified timing component. We have flagged this in Section 4.4 and Section 9.5 and again here, and readers should weigh it carefully when translating our recommendations into clinical practice or trial design.

Four further limitations follow from the design of this review and should be stated plainly. First, although the Section 3 describes a structured search, this work is not a systematic review, and we report neither record counts by stage nor a study-selection flow diagram, nor a list of included randomised trials by polyphenol class, so individual estimates cannot be traced to a defined pool of studies. Second, screening and extraction were not performed independently in duplicate and no formal risk-of-bias instrument was applied, which limits the reliability of study selection and appraisal. Third, we conducted no original quantitative synthesis, and every numerical estimate is reproduced from a published meta-analysis whose eligibility criteria, populations and exposure durations differ from those set out in Section 3.2, which introduces a mismatch between the evidence we describe and the evidence we quantify. Fourth, part of the mechanistic evidence invoked here derives from cell and animal work, from healthy participants, or from studies administering purified metabolites, and such evidence establishes plausibility rather than confirming a mechanism of dietary polyphenols in T2D.

We also note that the metabolite exposure framework applied here was set out by our group in a previous publication in Current Issues in Molecular Biology [8]. The conceptual overlap with that publication is substantial and deliberate, since the two-wave exposure model and its proposed signalling consequences were first developed there. The present manuscript is its clinical companion, and it differs in scope because it is restricted to human clinical evidence in adults with T2D and addresses trial design, exposure verification and the interpretation of heterogeneity rather than redox mechanism. Readers seeking the mechanistic derivation should consult that earlier paper, which is cited at each point where its content is summarised here.

## 11. Conclusions

Polyphenols are not pharmacological glucose-lowering drugs, and they are better regarded as dietary adjuncts whose effects are modest and whose pattern may be explicable by a biphasic metabolite exposure profile, in which host-derived conjugates act at the intestinal-hepatic interface within the first four hours while microbiome catabolites instead reach peripheral tissues between six and twenty-four hours later. This model offers a testable account of much of the apparent inconsistency in the literature, although it has not yet been validated against clinical endpoints. It proposes that effects remain modest because tissue exposure is intermittent and conjugate-mediated rather than pharmacological, and heterogeneous because metabotype, transporter genetics, matrix and timing all gate tissue access in different ways, and establishing whether this is so requires trials that measure both metabolite windows and prespecify the corresponding interaction analyses.

For clinical practice, anthocyanins, high-phenolic EVOO or purified hydroxytyrosol and oleuropein, and green tea catechins carry the strongest evidence as meal-timed adjuncts, best delivered in low-sugar, standardised formats. The realistic expectation for the best-supported class is a pooled HbA1c reduction of about 0.3% over a median of eight weeks, reported in trials that did not verify exposure, an effect that is modest and sits at the lower bound of clinical relevance, while alignment with earlier eating windows remains a research hypothesis rather than a clinical recommendation. Postprandial control and cardiometabolic markers respond more reliably and earlier than HbA1c itself, and cocoa flavanols and resveratrol are better positioned as cardiometabolic than as strictly glycaemic adjuncts. Tolerability at nutritional doses was generally good, on the basis of short trials in which adverse events were not systematically extracted, and this should not be taken as a formal safety conclusion.

For research, the agenda is clear. Investigators should test actual exposure, rather than nominal intake alone, and they should verify the relevant metabolites across both time windows described throughout this review. Stratification by metabotype should occur before randomisation takes place, and primary outcomes should be anchored in HbA1c measured at twelve weeks or more, together with CGM, adding hepatic imaging whenever MASLD coexists. Timing should be included as an experimental variable within factorial trial designs. The field is gradually moving from generic antioxidant claims towards precision nutrition, and the next steps are technically achievable, including identifying metabotype-defined responders, optimising formulation and timing, and combining polyphenols rationally with contemporary pharmacotherapy such as GLP-1RA and SGLT2i. With these elements in place, polyphenols may plausibly contribute a modest increment to glycaemic stability and cardiometabolic health in people living with T2D, although establishing whether that increment is reproducible will require trials that verify metabolite exposure and prespecify the corresponding interaction analyses.

## Figures and Tables

**Table 1:** Biphasic polyphenol metabolite exposure profile: early host-derived phase-II conjugates (0–4 h) and late microbiome-derived catabolites (6–24 h), with metabotype classification, non-producer prevalence, and mechanistic relevance to type 2 diabetes. N/A = not applicable (no metabotype split for host-enzyme-mediated conjugation). HT = hydroxytyrosol; EVOO = extra-virgin olive oil; MASLD = metabolic dysfunction-associated steatotic liver disease.

Catabolite/Metabolite	Dietary Precursor Class	Peak Plasma Window	Metabotype/Producer Status	Estimated Non-Producer Prevalence (Western Adults)	Mechanistic Relevance to T2D
**EARLY WAVE (0–4 h): Host-derived phase-II conjugates**					
Anthocyanin glucuronides & sulphates	Anthocyanins (berries, red/purple fruits)	1–2 h	Host enzymes (UGT1A, SULT1A); no metabotype split	N/A	Intestinal mucosal signalling (Keap1, NF-κB); local aglycone microbursts via β-glucuronidase; enterohepatic cycling
(−)-Epicatechin glucuronides & sulphates	Flavan-3-ols (cocoa, green tea, apples)	1–3 h	Host enzymes; no metabotype split	N/A	Endothelial NOS activation; eNOS-derived NO↑; mucosal glucose transport modulation
Trans-resveratrol glucuronide & sulphate	Stilbenes (grapes, wine, peanuts)	0.5–2 h	Host enzymes; variable first-pass	N/A	Sirtuin/SIRT1 activation; NF-κB dampening; low systemic aglycone
Hydroxytyrosol sulphate & glucuronide	Olive phenolics (EVOO, olives)	1–3 h	Host enzymes; no metabotype split	N/A	NRF2 activation; NF-κB suppression; lipotoxicity reduction; hepatic benefit in MASLD
**LATE WAVE (6–24 h): Microbiome-derived catabolites**					
Urolithin A-3-glucuronide (+urolithin B)	Ellagitannins (pomegranate, walnuts, berries)	8–12 h	Metabotype A (efficient); Metabotype B (partial); Non-producer	~25–40%	PINK1-Parkin mitophagy↑; TFEB/lysosomal biogenesis↑; AMPK activation; impaired in insulin-resistant skeletal muscle
5-(Hydroxyphenyl)-γ-valerolactone glucuronides	Flavan-3-ols & procyanidins (green tea, cocoa, grape seeds)	6–18 h	Variable colonic converter capacity; no formal metabotype classification yet	Not established; conversion variable	AMPK/MAPK activation; PPAR & AhR modulation; energy sensing; lipid metabolism; anti-inflammatory
S-Equol	Isoflavones (soy foods, daidzein)	8–24 h	Equol producer vs non-producer (microbiome-dependent)	~30–50% (Western); ~25–30% (Asian)	Oestrogen receptor β modulation; insulin sensitisation; endothelial effects; larger glycaemic benefit in producers
Dihydroresveratrol & lunularin	Stilbenes (resveratrol precursors)	6–16 h	Microbiome-dependent; variable across individuals	Not established	May contribute to anti-inflammatory effects attributed to resveratrol; poorly characterised in clinical trials
Phenylacid & hippurate derivatives	Multiple classes (ring fission products)	6–24 h	Non-specific; reflects overall microbiome catabolism	N/A	Index of colonic polyphenol engagement; less class-specific than urolithins or γ-valerolactones; complement targeted panels

**Table 2:** Summary of clinical evidence for dietary polyphenol classes in adults with type 2 diabetes. Dose ranges reflect the most commonly used regimens in published randomised controlled trials, and duration refers to the minimum intervention length at which the stated effect has been reliably observed. The final column reports the descriptive confidence label defined in Section 3.6, which refers to glycaemic primary endpoints unless otherwise noted and carries no GRADE equivalence. All entries are narrative summaries of the sources cited in the corresponding subsections of Section 5, and each row should be read together with that subsection. FMD = flow-mediated dilation; HOMA-IR = homeostasis model assessment of insulin resistance; HT = hydroxytyrosol; EVOO = extra-virgin olive oil; TG = triglycerides; oxLDL = oxidised low-density lipoprotein; iAUC = incremental area under the curve; MASLD = metabolic dysfunction-associated steatotic liver disease; CGM = continuous glucose monitoring; BP = blood pressure.

Polyphenol Class	Typical Dose	Min. Effective Duration	HbA1c Effect	Fasting Glucose Effect	Postprandial Effect	Vascular/Inflammatory Signal	Metabolite Verification Available	Descriptive Confidence (Section 3.6)
**Anthocyanins (berries)**	150–640 mg/day	≥12 weeks (HbA1c); ≥4 weeks (postprandial)	−0.31% pooled (Mao 2023 [36]); unverified exposure	−5 to −10 mg/dL	Consistent; reduced iAUC, improved time-in-range	hsCRP↓, IL-6↓, FMD↑; hepatic signals in MASLD	Yes (glucuronides/sulphates, 0–4 h; phenolic acids, 6–24 h)	Low-Moderate
**Stilbenes (resveratrol)**	250–1000 mg/day	≥12 weeks (HbA1c); inconsistent at <12 weeks	Inconsistent; variable across trials	Small, directional; inconsistent	Limited; some postprandial improvement	Strongest signal: hsCRP↓, TNF-α↓, FMD↑, MDA↓	Partial (glucuronides/sulphates); dihydroresveratrol rarely measured	Very Low-Low
**Catechins/Green tea (EGCG)**	300–800 mg/day catechins	≥12 weeks (HbA1c); ≥8 weeks (fasting glucose)	Small; mainly ≥12-week studies	−3 to −8 mg/dL; consistent	Improved when meal-timed; limited CGM data	FMD↑, hsCRP↓; modest BP reduction	Partial (epicatechin conjugates 0–4 h); γ-valerolactones rarely measured	Low-Moderate
**Cocoa flavanols/(−)-epicatechin**	200–600 mg/day total flavanols	≥8 weeks (vascular); ≥12 weeks (glycaemic)	Inconsistent; neutral in large trials	Variable; clearer in controlled mechanistic studies	Limited; confounded by confectionery matrix	Strongest signal: FMD↑, BP↓, hsCRP↓	Yes in mechanistic studies ((−)-epicatechin conjugates; γ-valerolactones)	Very Low-Low (glycaemic); Low-Moderate (vascular)
**Olive phenolics (HT, oleuropein, EVOO)**	HT 5–25 mg/day; oleuropein 50–100 mg/day; EVOO 20–50 mL/day	≥12 weeks (fasting/HbA1c); ≥4 weeks (postprandial)	Modest, inconsistent	Small; clearer with purified phenolics	Consistent reduction in postprandial glucose and insulin iAUC	FMD↑, hsCRP↓, TG↓, oxLDL↓; hepatic signals in MASLD	Minimal (HT conjugates rarely measured; late window absent)	Low
**Isoflavones (genistein, daidzein)**	40–100 mg/day	≥8–12 weeks	Inconsistent; larger in equol producers	Small; metabotype-dependent	Limited data	LDL↓ (modest); FMD↑ in postmenopausal women	S-equol measured in some trials; isoflavone conjugates rarely	Very Low
**Curcuminoids (curcumin)**	500–1500 mg/day ± enhancers	≥8 weeks (inflammation); HbA1c inconsistent	Inconsistent; limited by bioavailability	Occasional HOMA-IR improvement	Limited data	Most consistent: hsCRP↓, TNF-α↓, IL-6↓	Rarely verified; bioavailability-enhancer interactions uncharacterised	Very Low
**Ellagitannins (pomegranate, walnuts)**	Variable (juice 250–500 mL/day; extracts)	≥12 weeks; metabotype screening required	Modest or non-significant; no metabotype stratification in existing trials	Limited	Limited	Some anti-inflammatory signals; mechanistic (mitophagy) evidence in muscle	Urolithin A-3-glucuronide measured in some studies; late window required	Very Low

**Table 3:** Recommended metabolite verification panel for polyphenol randomised controlled trials in type 2 diabetes, organised by polyphenol class, sampling window, analytical method, and critical methodological notes. IS = internal standard; LC-MS/MS = liquid chromatography-tandem mass spectrometry; HT = hydroxytyrosol; EVOO = extra-virgin olive oil; EGCG = epigallocatechin gallate; γ-VL = γ-valerolactone; MUFA = monounsaturated fatty acids; 3,4-DHPEA-EDA = 3,4-dihydroxyphenylethanol-elenolic acid dialdehyde; O-DMA = O-desmethylangolensin.

Polyphenol Class	Key Analyte(s) to Verify	Sampling Window	Recommended Matrix	Analytical Method	Critical Notes
**EARLY WINDOW (0–4 h post-dose)—Phase-II conjugates**					
**Anthocyanins**	Cyanidin-3-glucuronide; peonidin-3-sulphate; delphinidin-3-glucuronide	Peak 1–2 h; collect 0.5, 1, 2, 4 h	Plasma + spot urine	LC-MS/MS with authentic standards	Sensitive to pH and light; stabilise with ascorbic acid immediately; report individual regioisomers, not aggregate sum
**Flavan-3-ols (green tea, cocoa)**	(−)-Epicatechin-3′-O-glucuronide; epicatechin-3′-sulphate; 3′-O-methyl-epicatechin-glucuronide	Peak 1–3 h; collect 0.5, 1, 2, 4 h	Plasma + spot urine	LC-MS/MS; stable-isotope IS required	Distinguish EGCG from epicatechin metabolites; decaffeinated extracts reduce matrix interference from methylxanthines
**Stilbenes (resveratrol)**	Trans-resveratrol-3-O-glucuronide; trans-resveratrol-3-sulphate	Peak 0.5–2 h; collect 0.5, 1, 2, 4 h	Plasma + spot urine	LC-MS/MS; protect from light	Free aglycone rarely detectable; conjugates dominate; also measure dihydroresveratrol in the late window as a microbiome catabolite
**Olive phenolics (HT, oleuropein)**	Hydroxytyrosol-sulphate; hydroxytyrosol-glucuronide; 3,4-DHPEA-EDA metabolites	Peak 1–3 h; collect 0.5, 1, 2, 4 h	Plasma + 24-h urine	LC-MS/MS; stabilise immediately	Distinguish from endogenous catecholamines; MUFA-matched comparator essential to isolate phenolic effects from EVOO matrix
**LATE WINDOW (6–24 h post-dose)—Microbiome-derived catabolites**					
**Ellagitannins (pomegranate, walnuts)**	Urolithin A-3-glucuronide; urolithin B-glucuronide	Peak 8–12 h; collect 6, 8, 12, 24 h + fasting trough	Plasma + spot or 24-h urine	LC-MS/MS; urolithin A-d4 IS	Metabotype pre-screening required; non-producers (~25–40%) yield undetectable urolithins despite adequate intake; classify and stratify before randomisation
**Flavan-3-ols/Procyanidins**	5-(3′,4′-Dihydroxyphenyl)-γ-valerolactone-glucuronide; 5-(4′-hydroxyphenyl)-γ-valerolactone-sulphate	Peak 6–18 h; collect 6, 12, 18, 24 h + fasting trough	Plasma + spot urine	LC-MS/MS; γ-VL-d5 IS preferred	No formal metabotype cut-points established; report quantitative AUC_6−24_h; individual conversion capacity varies substantially
**Isoflavones**	S-Equol; O-desmethylangolensin (O-DMA)	Peak 8–24 h; collect 8, 12, 24 h + fasting trough	Plasma + spot urine	LC-MS/MS or validated immunoassay; confirm S-stereoisomer	Equol-producer cut-point: >83 nmol/L plasma after standardised challenge; determine before randomisation and stratify arms accordingly
**All classes (non-specific panel)**	Hippuric acid; 3-hydroxyphenylacetic acid; 4-hydroxyhippuric acid; phenylacetic acid	6–24 h; include fasting trough	24-h urine (preferred) or spot urine	LC-MS/MS or LC-UV; normalise to creatinine	Index of overall colonic polyphenol catabolism; complement targeted panels to confirm microbiome engagement even when class-specific catabolites are absent

**Table 4:** Principal drug–nutrient interaction signals for dietary polyphenol classes in type 2 diabetes, organised by interaction category. The final column, added at review, states the type of evidence, the population in which it was obtained and the resulting certainty for each row, and it should be read together with the caveat given in Section 8.2. Rows supported only by in vitro or mechanistic evidence are not clinical interaction findings. CYP = cytochrome P450; P-gp = P-glycoprotein (ABCB1); BCRP = breast cancer resistance protein (ABCG2); INR = international normalised ratio; TI = therapeutic index; GLP-1RA = glucagon-like peptide-1 receptor agonist; DPP-4i = dipeptidyl peptidase-4 inhibitor; SGLT2i = sodium-glucose cotransporter-2 inhibitor; AMPK = AMP-activated protein kinase; CKD = chronic kidney disease; eGFR = estimated glomerular filtration rate; MASLD = metabolic dysfunction-associated steatotic liver disease; ACE = angiotensin-converting enzyme; ARB = angiotensin receptor blocker; TSH = thyroid-stimulating hormone; EGCG = epigallocatechin gallate.

Polyphenol Class	Drug or Drug Class	Mechanism of Interaction	Clinical Relevance	Practical Recommendation	Evidence Type, Population and Certainty
**CYP450 and Transporter Interactions**					
**Resveratrol; concentrated phenolics**	Warfarin; acenocoumarol	CYP2C9 inhibition → reduced warfarin clearance; potential additive antiplatelet effect	Moderate: INR elevation in case reports; bleeding risk	Avoid resveratrol > 500 mg/day with oral anticoagulants; monitor INR if co-administered; inform anticoagulation clinic	Case reports plus in vitro CYP2C9 inhibition. No randomised interaction study and no data in T2D. Theoretical signal, very low certainty [89].
**Resveratrol; quercetin; kaempferol**	Statins (simvastatin, atorvastatin)	CYP3A4 and P-gp inhibition → increased statin plasma exposure; myopathy risk at higher phenolic doses	Low–moderate: mainly relevant at high supplement doses	Use nutritional doses; avoid high-dose supplements with myopathy-risk statins; monitor for muscle symptoms	In vitro and animal CYP3A4 and P-gp inhibition. No human interaction study at nutritional doses. Preclinical signal, very low certainty [89].
**Curcuminoids (+ piperine)**	Tacrolimus; cyclosporin; midazolam; narrow-TI drugs	Piperine inhibits CYP3A4 and P-gp → increases systemic exposure of co-administered drugs; curcumin inhibits CYP1A2, CYP2C9	Moderate–high, with immunosuppressants or narrow-TI drugs	Avoid piperine-enhanced curcumin with narrow-TI drugs; separate doses by ≥2 h minimum; disclose supplement use to prescriber	Human pharmacokinetic studies in healthy volunteers. No data in adults with T2D. Moderate certainty for the pharmacokinetic effect, unknown clinical consequence [90].
**Green tea (EGCG)**	Bortezomib (proteasome inhibitor)	EGCG may antagonise bortezomib via covalent binding; demonstrated in vitro; dietary doses low risk	Low at dietary intake; potentially significant with high-dose EGCG supplements in oncology	Avoid high-dose EGCG supplements in patients receiving bortezomib-based regimens	In vitro oncology cell models only, with no human confirmation. Preclinical signal, very low certainty.
**Olive phenolics; anthocyanins**	BCRP substrates (rosuvastatin, methotrexate, sulfasalazine)	BCRP (ABCG2) inhibition → reduced efflux → increased plasma substrate exposure	Low at dietary doses; theoretically relevant with concentrated extracts	Dietary amounts unlikely to cause clinically significant interaction; monitor for substrate toxicity if concentrated extracts are used alongside BCRP substrates	In vitro transporter inhibition and mechanistic inference. No human interaction study. Very low certainty at dietary intake.
**Interactions with Antidiabetic Therapies**					
**All classes (postprandial-active)**	GLP-1 receptor agonists; DPP-4 inhibitors	GLP-1RAs slow gastric emptying → delayed polyphenol absorption and shifted conjugate peak; shared postprandial glucose-lowering may mask or amplify phenolic effects	Low clinical risk; interpretive problem for trials	Document and balance GLP-1RA/DPP-4i dose across arms; treat as a prespecified stratification variable; interpret postprandial CGM data in this context	Mechanistic inference from established gastric-emptying pharmacology. No dedicated interaction study. Trial-design consideration rather than a clinical interaction.
**All classes**	SGLT2 inhibitors	SGLT2i lower fasting and postprandial glucose independently; may mask small polyphenol glycaemic signals; additive renal glucose excretion possible	Low clinical risk; relevant for trial interpretation	Balance across arms; prespecify subgroup by SGLT2i use; run sensitivity analyses in SGLT2i users vs non-users	Mechanistic inference. No interaction study. Trial-design consideration rather than a clinical interaction.
**Flavan-3-ols; anthocyanins**	Metformin	Metformin modulates gut microbiome and AMPK; may synergise with late-window catabolites (AMPK activation); additive effects plausible	Low risk; possibly beneficial interaction	Document metformin dose and duration; consider as an effect modifier in subgroup analysis	Mechanistic and preclinical inference. No human interaction study. Very low certainty.
**Microbiome-Mediated and Other Interactions**					
**Ellagitannins; flavan-3-ols; isoflavones**	Antibiotics (any class)	Broad-spectrum antibiotics deplete urolithin-, γ-valerolactone-, and equol-producing bacteria; late-wave catabolite generation eliminated for 4–8 weeks or longer	High for late-window metabolite studies; urolithin and equol producers may become temporary non-producers	Exclude recent antibiotic use (within 8 weeks) from eligibility or treat as covariable; re-screen metabotype after any >4-week course during trial	Human microbiome and metabolomic studies, mostly in healthy volunteers. Well supported for loss of catabolite output, untested for clinical outcome. Low to moderate certainty.
**All classes (late-window dependent)**	Proton pump inhibitors (omeprazole, lansoprazole)	Raised gastric pH alters polyphenol stability and absorption kinetics; PPI-driven microbiome changes reduce late-window catabolite generation	Moderate: blunts late-window signal; underappreciated confounder in polyphenol trials	Document PPI use; prespecify as stratification factor in trials targeting late-window effects; sensitivity analysis excluding PPI users	Observational microbiome data plus mechanistic inference. No controlled interaction study. Low certainty.
**Green tea; cocoa; anthocyanins**	Iron supplements; iron-rich meals	Polyphenol chelation of non-haem iron reduces absorption by roughly 50 to 90% depending on the polyphenol dose and on concurrent intake	Moderate: clinically relevant in iron-deficient patients or those with MASLD-related anaemia	Separate polyphenol intake from iron supplementation by ≥2 h; administer iron in the morning and polyphenols with lunch or the evening meal	Human stable-isotope absorption studies in healthy volunteers. Well supported for non-haem iron absorption, not studied in T2D. Moderate certainty [91].
**High-volume berry or pomegranate juice**	Potassium-sparing diuretics; ACE inhibitors; ARBs in advanced CKD	High potassium content of fruit juices may worsen hyperkalaemia in impaired renal function	Moderate–high in advanced CKD (eGFR < 30 mL/min/1.73 m^2^)	Prefer capsules or standardised extracts over whole juices in advanced CKD or with renin-angiotensin blockade	Food-composition data plus general CKD nutrition guidance. No polyphenol-specific study. Precautionary, low certainty.
**Soy isoflavones**	Levothyroxine	Soy reduces levothyroxine absorption; potential thyroid interference with chronic high-dose isoflavone intake	Low–moderate: mainly relevant with high-dose supplements, not standard dietary soy	Separate levothyroxine from soy meals or supplements by ≥4 h; monitor TSH in patients starting isoflavone supplementation	Human absorption studies and case series. Established for levothyroxine absorption, uncertain for thyroid outcome. Low to moderate certainty.

**Table 5:** Current clinical guidance compared with research recommendations for dietary polyphenols in type 2 diabetes, organised by decision domain. Entries in the clinical column are supported by human randomised evidence of at least modest quality in adults with T2D or prediabetes, whereas entries in the research column rest on mechanistic reasoning, indirect evidence or preliminary data and are offered as trial-design priorities. CGM = continuous glucose monitoring; LC-MS/MS = liquid chromatography-tandem mass spectrometry; EGCG = epigallocatechin gallate; CKD = chronic kidney disease; MASLD = metabolic dysfunction-associated steatotic liver disease.

Decision Domain	Current Clinical Practice: What Can Reasonably Be Recommended Now	Research Recommendation: Not Yet Ready for Routine Practice
**Choice of polyphenol class**	Anthocyanin-rich berries, high-phenolic extra-virgin olive oil or purified hydroxytyrosol, and green tea catechins, offered as dietary adjuncts alongside standard therapy.	Direct metabolite formulations such as urolithin A, and class selection guided by an individual metabotype profile.
**Expected magnitude of effect**	A pooled HbA1c reduction of about 0.3% for anthocyanins over a median of eight weeks, with postprandial and vascular gains appearing earlier and more reliably.	Whether verified metabolite exposure yields a larger effect than the unverified pooled estimate, which no existing trial can answer.
**Formulation and matrix**	Low-sugar beverages or standardised capsules in preference to high-sugar juices, and decaffeinated preparations where caffeine sensitivity is a concern.	Microencapsulation, sustained-release systems, and emulsified vehicles designed to retime exposure between the early and late windows.
**Timing of intake**	Taking the polyphenol with a meal rather than fasted, which rests on established absorption and first-pass pharmacokinetics.	Alignment with the first main meal of the day or with an earlier eating window, which rests on indirect chrononutrition evidence and requires a factorial timing trial.
**Metabotype screening**	No routine screening is indicated, and treatment decisions should not depend on producer status at present.	Pre-randomisation classification of urolithin, gamma-valerolactone and equol producer status, with stratified randomisation and prespecified interaction testing.
**Metabolite verification**	Not available or indicated in routine care, and adherence should be assessed by conventional means.	Targeted LC-MS/MS panels sampling both the 0 to 4 h and the 6 to 24 h windows, reported as a primary pharmacokinetic outcome.
**Monitoring of response**	Continuous glucose monitoring where already available, used to document individual postprandial response and to set realistic expectations.	Prespecified CGM endpoints including time-in-range and postprandial time-above-range, powered against a defined minimally important difference.
**Safety and interactions**	Nutritional doses, avoidance of high-dose fasted EGCG, attention to anticoagulant and narrow-therapeutic-index co-medication, and caution with high-volume fruit drinks in advanced CKD.	Systematic characterisation of interaction risk for concentrated extracts and bioavailability-enhanced formulations, which is currently absent.
**Population most likely to benefit**	Adults with prominent postprandial hyperglycaemia, HbA1c above target, coexisting MASLD, or impaired vascular function.	Formal identification of responder phenotypes through individual participant data analysis, which has not yet been performed.

## Data Availability

No new data were created or analyzed in this study. Data sharing is not applicable to this article.
